# Determinants of RPA megafoci localization to the nuclear periphery in response to replication stress

**DOI:** 10.1093/g3journal/jkac116

**Published:** 2022-05-14

**Authors:** Seong Min Kim, Susan L Forsburg

**Affiliations:** Molecular & Computational Biology, University of Southern California, Los Angeles, CA 90007, USA; Molecular & Computational Biology, University of Southern California, Los Angeles, CA 90007, USA

**Keywords:** Replication stress, MCM4, checkpoint, RPA, DNA repair

## Abstract

Upon replication stress, ssDNA, coated by the ssDNA-binding protein RPA, accumulates and generates a signal to activate the replication stress response. Severe replication stress induced by the loss of minichromosome maintenance helicase subunit Mcm4 in the temperature-sensitive *Schizosaccharomyces pombe* degron mutant (*mcm4-dg*) results in the formation of a large RPA focus that is translocated to the nuclear periphery. We show that resection and repair processes and chromatin remodeler Swr1/Ino80 are involved in the large RPA foci formation and its relocalization to nuclear periphery. This concentrated accumulation of RPA increases the recruitment of Cds1 to chromatin and results in an aberrant cell cycle that lacks MBF-mediated G1/S accumulation of Tos4. These findings reveal a distinct replication stress response mediated by localized accumulation of RPA that allows the evasion of cell cycle arrest.

## Introduction

DNA replication progression can be disrupted by intrinsic stress (e.g. caused by replisome mutations or fragile DNA) or extrinsic stress (e.g. caused by treatment with genotoxins). The resulting DNA damage is managed by diverse repair processes to promote completion of DNA synthesis and faithful chromosome segregation (rev in Cortez 2019). Work in a number of different systems has visualized the formation of DNA repair foci in live cells undergoing replication stress. Accumulation of single-strand DNA (ssDNA), marked by ssDNA-binding protein RPA, is a key sign of genotoxic stress and is managed tightly to ensure genome integrity. Causes of ssDNA include fork regression, resection, helicase uncoupling, and D-loop and R-loop formation (rev in Sabatinos and Forsburg 2015; Toledo *et al.* 2017). Recruitment of RPA to ssDNA at stressed replication forks helps initiate DNA repair and checkpoint responses (Osborn *et al.* 2002; Zou and Elledge 2003; Yin *et al.* 2021) and RPA is a limiting mediator of replication stress response (Toledo *et al.* 2013; Ercilla *et al.* 2020).

The nuclear periphery has been shown to be an important hub that facilitates the repair of various DNA damages such as heterochromatin breaks, telomeric lesions and failed replication forks (rev in Freudenreich and Su 2016; Whalen and Freudenreich 2020). Distinct pathways are involved in the localization of repair foci to the periphery. For example, SUMOylation of repair proteins involved in homologous recombination (HR) regulates localization of double-strand breaks (DSB) within heterochromatin and failed replication forks to nuclear pore complex (NPC) (Ryu *et al.* 2015; Kramarz *et al.* 2020; Whalen *et al.* 2020, rev. in Schirmeisen *et al.* 2021). Other observations implicate ATP-dependent chromatin remodeler complexes Swr1 and Ino80, which are involved in regulation of transcription, replication, and repair (rev in Poli *et al.* 2017); these also promote DSB localization to an essential inner nuclear membrane (INM) Sad1-Unc-84-related (SUN) domain protein Mps3 in budding yeast (Horigome *et al.* 2014). The localization to the nuclear periphery by any mechanisms may allow more efficient concentration of repair proteins to promote efficient repair and maintain genome stability.

The minichromosome maintenance (MCM) helicase comprises six subunits and is responsible for replication initiation and progression (rev. in Burgers and Kunkel 2017). Mutations in Mcm4, one of the subunits of the MCM complex, have been implicated in various cancers and are known to induce chromosomal abnormalities such as micronuclei formation, DSB, and chromosome rearrangement (Shima *et al.* 2007; Giaginis *et al.* 2010; Bagley *et al.* 2012; Casey *et al.* 2012; Gineau *et al.* 2012; Hughes *et al.* 2012; Sabatinos *et al.* 2015; Kim and Forsburg 2020). Conditional mutations in *Schizosaccharomyces pombe mcm4* have different phenotypes and have been useful in investigating replication stress and its responses (Coxon *et al.* 1992; Lindner *et al.* 2002; Nitani *et al.* 2008; Sabatinos *et al.* 2015; Ranatunga and Forsburg 2016; Kim and Forsburg 2020). Previously, we showed that different alleles of *mcm4* have different patterns of RPA repair foci (Sabatinos *et al.* 2015; Kim and Forsburg 2020). Specifically, the canonical *mcm4-ts* allele (*mcm4-M68*; Nasmyth and Nurse 1981), which is associated with late S phase failure, accumulation of DSB, and successful activation of the DNA damage checkpoint, generates dispersed puncta of RPA in the nucleus (Bailis *et al.* 2008; Sabatinos *et al.* 2015; Kim and Forsburg 2020). Conversely, the *mcm4-dg* allele isolated in (Lindner *et al.* 2002), has a profound DNA replication defect but fails to activate Chk1, continues to divide, and forms a single large “megafocus” of RPA that localizes to the nuclear periphery (Sabatinos *et al.* 2015; Kim and Forsburg 2020). A key difference between these alleles is that *mcm4-ts*, but not *mcm4-dg*, activates the endonuclease Mus81, generating breaks that activate the damage checkpoint and promote cell cycle arrest (Sabatinos *et al.* 2015; Kim and Forsburg 2020).

In this study, we investigate the factors required for the formation of the megafocus and its localization. We show that diverse pathways involved in resection, DNA repair, and replication fork protection are crucial for coalescence of small multifoci RPA into megafoci RPA in *mcm4-dg*. Loss of these proteins results in increased multicluster RPA and restoration of phosphorylated H2A, suggesting increased break formation. The chromatin remodeler Swr1/Ino80 pathway, but not sumoloylation, is involved in the coalescence and translocation of RPA foci to the nuclear periphery. Both non-homologous end-joining (NHEJ) and HR repair proteins are recruited to RPA foci, hinting at various types of DNA lesions following replication stress. We observe that *mcm4-dg* strain also shows increased Cds1 association with RPA that correlates in aberrant cell division. These findings suggest that the distinctive RPA foci pattern in *mcm4-dg* indicates a unique form replication stress that results in evasion of proper cell cycle arrest and demonstrate that RPA foci pattern may be a useful tool in assessing different types of DNA damage responses.

## Materials and methods

### Yeast strains and media

*S.* *pombe* strains (Supplementary Table 1) were cultured using standard protocols and media (Sabatinos and Forsburg 2010), grown in supplemented Edinburgh minimal medium (EMM) and incubated at 36°C for 4 h for imaging. For viability, asynchronous cultures were placed at 36°C for 4 h, and aliquots with equivalent numbers of cells were plated on YES plate, and grown at 25°C. Number of colonies grown after 36°C pulse were normalized to number of colonies grown from the initial culture.

### Chromosome loss and rearrangements

Strains with ChL minichromosome were grown as in Nakamura *et al.* (2008), Li *et al.* (2013), and Sabatinos *et al.* (2015). Cultures were incubated at 36°C for 4 h and then plated on YES and grown at 25°C. Colonies were then replica-plated and assessed for Leu−, Ade−, and hygromycin status. Minichromosome loss rate was calculated from the number of Leu− Ade− cells. Gross chromosomal rearrangement (GCR) rate was calculated from the number of Leu+ Ade− and Ade− hygromycin resistant cells. Both mutation rates were determined by fluctuation analysis based on the Lea–Coulson method (Foster 2006).

### Microscopy

General protocols were as described by Green *et al.* (2009). Briefly, cells cultured in supplemented EMM were placed on 2% agarose pads sealed with VaLaP (1/1/1 [wt/wt/wt] Vaseline/lanolin/paraffin) for live-cell imaging. Images were acquired using a DeltaVision microscope (with softWoRx version 4.1; GE, Issaquah, WA) using a 60× (NA 1.4 PlanApo) or 100× (1.35, U Plan Apo, PSF, IX70) lens, solid-state illuminator, and 12-bit CCD camera. Images were deconvolved and maximum intensity projected for fluorescence images (sofrWoRX) and transmitted light images were inverted and added for outline of the cells (ImageJ) (Schindelin *et al.* 2012). Static images were projected from seven z-stacks of 0.5 µm with 0.08–0.5 s exposure time. For timelapse imaging, cells were maintained in a chamber with constant temperature (36°C) and imaged with shorter exposure time (0.08 s) and four z-stacks to reduce phototoxicity.

### Immunoprecipitation

Spheroplasts were prepared in PEMS buffer with 1 mg/ml lysing enzymes (Sigma L1412) and 1 mg/ml Zymolase 100T as in Kunoh and Habu (2014). Spheroplasts from RPA-CFP and Cds1-myc containing cells were lysed with ice-cold lysis buffer and lysates were separated by centrifugation to chromatin-bound fraction or chromatin un-bound fraction. Resulting fractions were incubated with GFP-Trap magnetic agarose beads (Chromotek) at 4°C. Agarose beads were then resuspended in 100 µl 2× SDS sample buffer (8% SDS, 120 mM Tris-HCl, pH 6.8, 10% glycerol, 10% 2-mercaptoethanol, 0.02% bromophenol blue) and boiled at 95°C for 5 min. Eluted supernatant was analyzed in SDS-PAGE/Western blot.

### Western blot

Cells in supplemented EMM were collected and whole-cell protein extract was prepared by vortexing acid-washed glass beads in 20% trichloroacetic acid (TCA) and washing beads with 5% TCA. Lysates were boiled for 5 min in Laemmli Sample buffer (4% SDS, 60 mM Tris-HCl, pH 6.8, 5% glycerol, 5% 2-mercaptoethanol, 0.01% bromophenol blue) and analyzed by SDS-PAGE. Primary antibodies used were as follows: anti-phospho-H2A (Abcam ab17353; 1:1,000), anti-H2A (Cell Signaling 3636S; 1:1,000; re-probed after phospho-H2A), anti-GFP (Abcam ab291; 1:1,000), anti-myc (Abcam ab9106; 1:1,000) anti-PCNA (Santa Cruz sc-56; 1:1,000), and anti-cdc2 (Abcam ab5467; 1:1,000). After secondary antibody (Alexa Flour 488 or 647; 1:6,000) incubation, blots were developed using Amersham Typhoon biomolecular imager. Intensity of bands were quantified with ImageJ.

### Statistical analysis

Mean ± SEM are shown on quantifications for % multifoci cells, RPA size, and cell length. For minichromosome loss rate and GCR rate, center line indicates median, box indicates the 25th and 75th percentiles, and error bars extend 1.5 times the interquartile range from the 25th and 75th percentiles. Boxplot was generated using BoxPlotR according to Spitzer *et al.* (2014). Significance was determined by Student’s *t*-test with a two-tailed distribution: *P < 0.05, **P < 0.01, ***P < 0.001, n.s.: not significant.

## Results

### Megafocus RPA is localized to the nuclear periphery

**Fig. 1.**
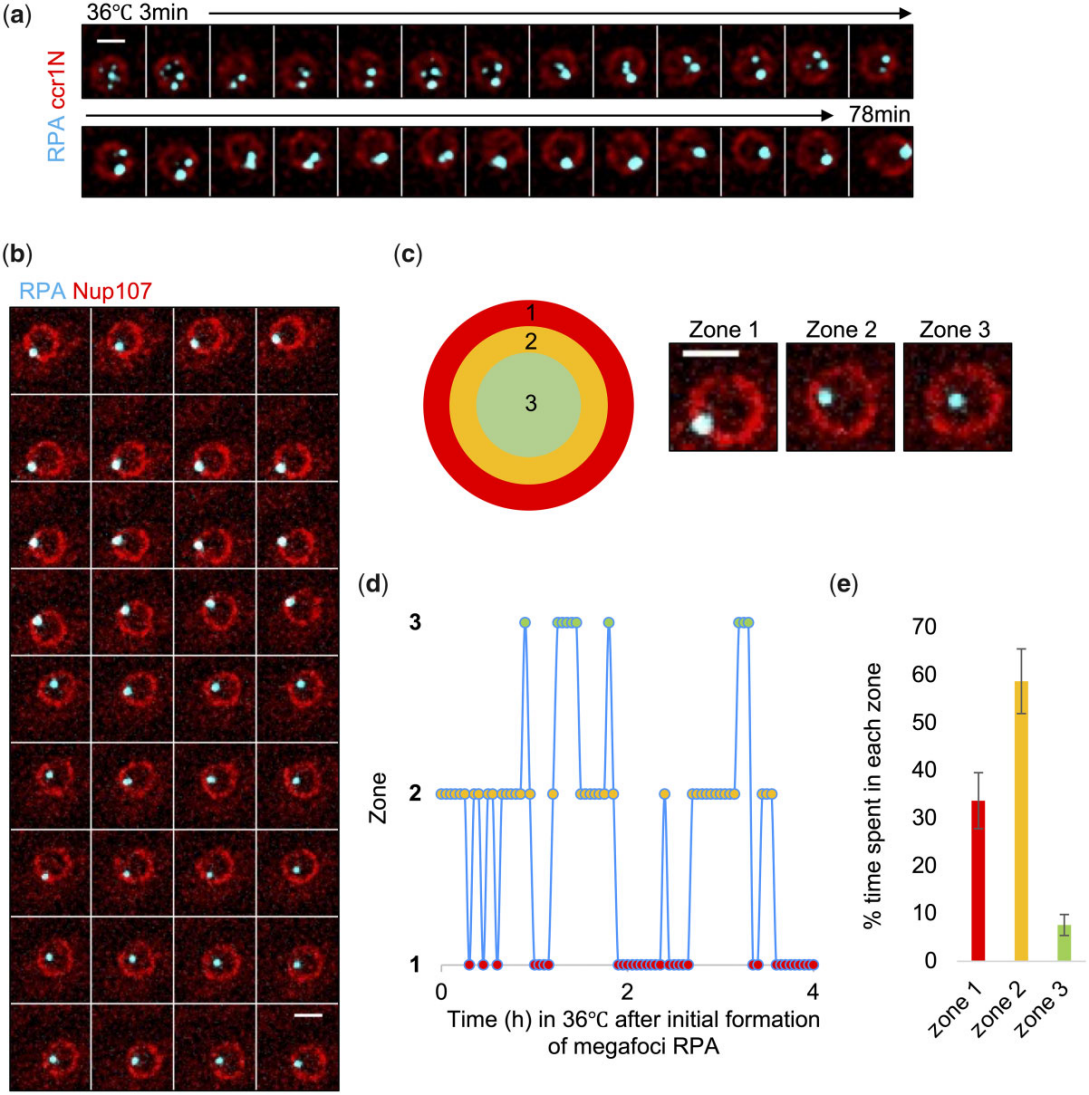
Replication stress in *mcm4-dg* induces accumulation of RPA that relocalizes to nuclear periphery. a) *mcm4-dg* nucleus imaged over time for RPA-CFP and ccr1N-mCherry at 36°C (FY7423). b) Left, *mcm4-dg* nucleus imaged as in (a) after megafoci RPA formed (FY9447). c) Nuclear space divided into 3 zones to score RPA position: zone 1 (nuclear periphery), zone 2 (near nuclear periphery), and zone 3 (near center of nucleus). d) Changes in RPA location (zone) over time at 36°C. e) Percent time megfoci RPA spent in each zone (*n* = 5). Scale bar 2 µm.

Previously, we showed that *mcm4-dg* cells, when placed at the restrictive temperature, form transient multifoci RPA near the center of the nucleus that subsequently merge into a large single focus (megafocus) at the nuclear periphery (Kim and Forsburg 2020) (Fig. 1a). To investigate whether the megafocus RPA remains stably associated at the nuclear periphery, strains were imaged every 3 min for 4 h at the restrictive temperature and RPA megafocus location relative to nuclear membrane marker ccr1N was assessed (Fig. 1, b–e). Zone 1 indicates overlap between RPA and ccr1N; zone 2 indicates RPA near or partially touching ccr1N; Zone 3 indicates RPA position near the center of the nucleus. Figure 1, b–d shows a representative depiction of the RPA megafocus position in a typical cell and Fig. 1e is a summarized quantification of % time megafoci RPA spent in each zone. We observe that the megafocus RPA is not static and does move to the nuclear center occasionally but spends most of the time on or near the nuclear periphery.

### Recruitment of damage response proteins to megafocus

**Fig. 2.**
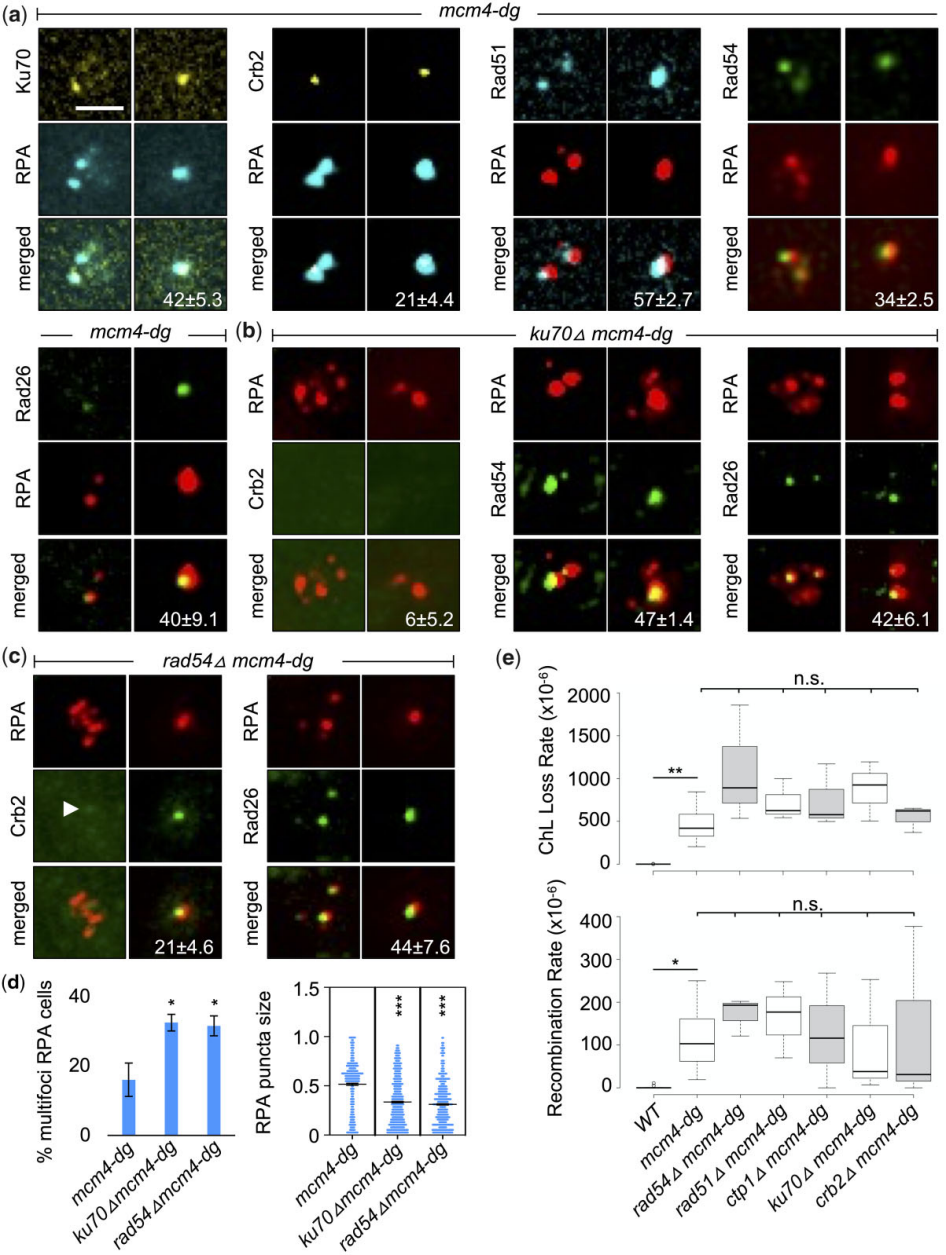
Repair proteins are recruited to RPA foci in *mcm4-dg* but do not impact chromosome loss or recombination rate. a) *mcm4-dg* nuclei were imaged for RPA-CFP or RPA-mCherry with Ku70-YFP, Crb2-YFP, Rad51-CFP, Rad54-GFP, or Rad26-GFP after 1–4 h at 36°C (FY9405, FY9639, FY10018, FY9945, FY9894). Representative multifoci RPA and megafoci RPA images are shown. Numbers on the bottom images indicate % RPA that colocalizes with the repair protein (mean ± SE). b) Ku70-deficient *mcm4-dg* cells imaged for RPA-mCherry and Crb2-GFP, Rad54-GFP, or Rad26-GFP. *ku70△mcm4-dg* cells mostly have multifoci RPA that lack Crb2 foci but have Rad54 or Rad26 foci (FY10003, FY10010, FY10006). c) Rad54-deficient *mcm4-dg* cells imaged for RPA-mCherry and Crb2-GFP or Rad52-GFP. *rad54△mcm4-dg* cells have multifoci RPA or small single RPA that have Crb2 and Rad26 foci (FY10021, FY10007). Arrowhead denotes a faint Crb2 foci. Scale bar 2 µm. d) Percent of cells containing multifoci RPA and individual RPA puncta size measured using ImageJ from images as in (b) and (c). e) Strains with ChL minichromosome were incubated at 36°C for 4 h and plated on YES plates (FY4003, FY9431, FY10025, FY10029, FY10031, FY10033, FY10035). Replica-plated colonies on Leu-, Ade-, and hygromycin were assessed for chromosome loss and recombination rate. See *Methods* for details. *P < 0.05, **P < 0.01, ***P < 0.001, n.s. not significant.

Translocation to the nuclear periphery is associated with DNA damage repair in many systems (Lemaître *et al.* 2012; Horigome *et al.* 2014; Ryu *et al.* 2015; Kramarz *et al.* 2020; Lamm *et al.* 2021). We investigated whether additional proteins involved in DNA damage response or repair associate with RPA megafoci. As previously observed, the DNA-end binding protein Ku70 is associated with some multifoci RPA in *mcm4-dg* and persists with the megafoci RPA (Kim and Forsburg 2020) (Fig. 2a). HR proteins Rad51 and Rad54 were also found colocalized with RPA foci (Fig. 2a), consistent with previous observations of Rad52 (Sabatinos *et al.* 2015). We also observe recruitment of the checkpoint proteins Rad26 (HsATRIP), which is associated with RPA (Namiki and Zou 2006), and Crb2 (Hs53BP1), which is associated with specific histone modifications at DNA ends (Hsiao and Mizzen 2013).

Next, we examined the co-dependence of recruitment of these factors. Loss of Ku70 resulted in increased multifoci RPA (Kim and Forsburg 2020) and these cells failed to recruit Crb2 to RPA foci (Fig. 2, b and d). Interestingly, both of these proteins are linked to NHEJ (rev in Daley and Sung 2014; Marini *et al.* 2019), which suggests that active NHEJ or end-processing may be linked to processing multifoci into the megafocus, and subsequent localization to the periphery. In contrast, checkpoint regulator Rad26 (which binds RPA; Namiki and Zou 2006) and the HR protein Rad54 are not dependent upon Ku (Fig. 2b).

Loss of Rad54 resulted in increased multifoci RPA as well as small single RPA foci but did not affect Crb2 or Rad26 recruitment to RPA foci, although Crb2 foci were much fainter in multifoci RPA (Fig. 2, c and d). This is consistent with Rad54 acting downstream of the checkpoint responders and also implicates HR in the formation of the megafocus. Crb2 deficiency resulted in small single RPA foci while Rad26 deficiency resulted in increased multifocus RPA formation (Supplementary Fig. 1, a and b) but neither Crb2 nor Rad26 affected the recruitment of repair proteins to RPA (Supplementary Fig. 1, c and d).

We know that following a pulse at the restrictive temperature, *mcm4-dg* survivors have increased rates of chromosome loss and gross chromosome rearrangement (GCR) compared to WT or *mcm4-ts* cells (Sabatinos *et al.* 2015). We examined whether loss of HR or NHEJ proteins resulted in an increase in chromosome loss rate, but the effect was not statistically significant (*P*-value >0.1) (Fig. 2e). Deletion of *rad51* or *rad54* slightly increased GCR, while deletion of *ku70* or *crb2* had a slightly reduced GCR compared to the single *mcm4-dg* mutant, but again, these were not statistically significant. Thus, recruitment of repair proteins and formation of the megafocus are not strongly correlated with chromosome loss or GCR outcomes.

### Megafocus formation depends on damage processing proteins

We previously demonstrated that downregulation of the structure-specific endonuclease Mus81 by the replication checkpoint kinase Cds1, and associated failure to form DNA DSB, correlates with the formation of megafoci RPA in *mcm4-dg* cells, and evasion of the Chk1 damage checkpoint (Sabatinos *et al.* 2015; Kim and Forsburg 2020). This suggests that the megafocus forms in response to particular structures or lesions that remain unprocessed in the absence of Mus81.

We asked if other proteins that are known to contribute to processing of replication forks or damaged DNA also correlate with RPA megafocus formation. Exonuclease Exo1 has 5′ to 3′ exonucleolytic resection activity at the site of a stalled replication fork (Saada *et al.* 2017). DNA repair protein Fan1 has both 5′ to 3′ exonuclease and 5′-flap endonuclease activity (Jin and Cho 2017). RecQ type DNA helicase Rqh1 regulates recombinational repair of DNA lesions occurring during DNA replication (Willis and Rhind 2009). Fbh1 is a F-box helicase that regulates Rad51-dependent HR by helicase activity and as a part of ubiquitin ligase complex (Tsutsui *et al.* 2014). Mrc1, Swi1, and Swi3 are components of the replication fork protection complex (FPC) that stabilizes stalled replication forks to limit accumulation of ssDNA, and also contribute to in Cds1 activation (Noguchi *et al.* 2004; Shimmoto *et al.* 2009). Mrc1 has two Rad3/Tel1 target TQ sites, T645 and T653, which when mutated, specifically fail to activate Cds1 without impacting fork processivity (Xu *et al.* 2006).

**Fig. 3.**
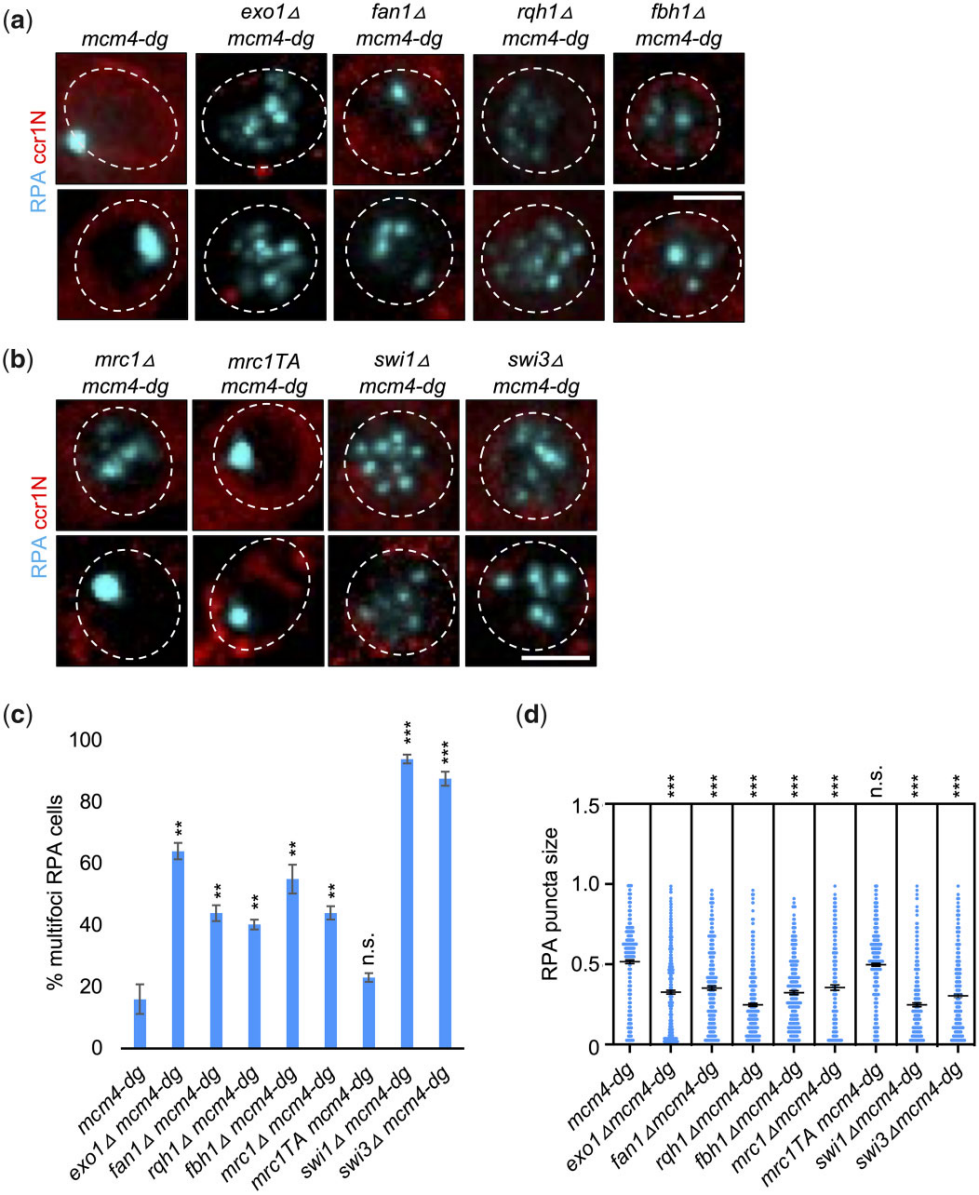
Repair proteins and resection enzymes contribute to megafoci RPA formation. a, b) Nuclei of indicated strains imaged for RPA-CFP and ccr1N-mCherry after 4 h at 36°C (FY7423, FY8770, FY9884, FY9705, FY9926, FY9939, FY9951, FY9987, FY9989). c) Percent of cells containing multifoci RPA from images as in (a) and (b). d) Individual RPA puncta size measured using ImageJ from images as in (a) and (b). Scale bar 2 µm. **P < 0.01, ***P < 0.001, n.s. not significant.

We observed that deletion of Exo1, Fan1, Rqh1, Fbh1, and FPC components all reduced RPA megafoci in *mcm4-dg* cells (Fig. 3, a–c), leading to increased multifoci. Correspondingly, presence of multifoci corresponded to decreased size of individual RPA puncta (Fig. 3d). This effect was particularly striking for the FPC mutants Swi1 and Swi3, and the long-range exonuclease Exo1. Notably, megafocus RPA in Mrc1 T645A T653A (*mrc1TA mcm4-dg*) was similar to the *mcm4-dg* single mutant, indicating that Mrc1’s role in the FPC and fork processivity, rather than as a Cds1 activator, is necessary for the RPA phenotype observed in *mcm4-dg* cells (Fig. 3, b–d). Together, these implicate DNA processing in formation of the megafocus.

### Megafocus formation inversely correlates with DNA DSB signal

**Fig. 4.**
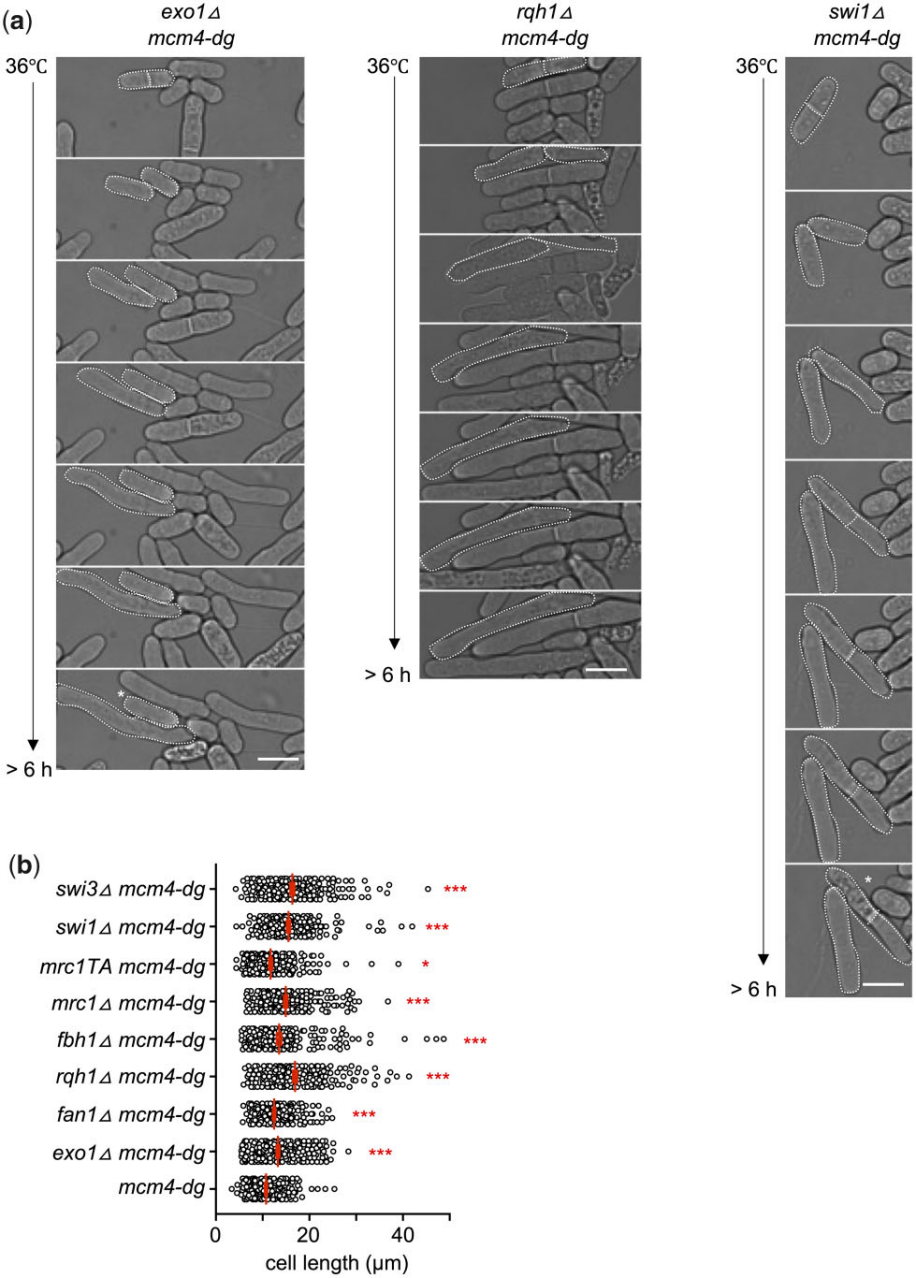
Defects in megafoci RPA formation (increase in multifoci RPA) is correlated with increased cell cycle arrest indicated by cell elongation at restrictive temperature. a) Examples from timelapse images of double mutants that increased multifoci RPA at 36°C (Fy8770, FY9705, FY9987 shown). * denotes un-dividing or dying cells. Scale bar 10 µm. b) Cell length measured from cells imaged as in (a) after 4 h at 36°C. *P < 0.05, ***P < 0.001.

The canonical temperature-sensitive *mcm4-ts* has multifoci RPA and also forms Mus81-dependent DNA DSBs, which activate the DNA damage checkpoint and correlate with phosphorylation of the histone H2A and cell elongation and arrest; these are not observed in *mcm4-dg* (Bailis *et al.* 2008; Sabatinos *et al.* 2015; Kim and Forsburg 2020). We asked whether the increased multifocus clusters observed in the double mutants between *mcm4-dg* and repair genes (Fig. 3) correspond with a DNA DSB response, measured by Western blotting of phospho-H2A and there was a trend toward increased phospho-H2A especially in cells lacking FPC (Supplementary Fig. 2). Presence of increased phospho-H2A also correlates with an increase of cell length, and reduced division (Fig. 4, a and b). We infer that the double mutants increase DNA DSBs resulting in Chk1 activation, multifocus RPA formation, and cell cycle arrest.

### Identifying pathways required for localization of megafocus to periphery

**Fig. 5.**
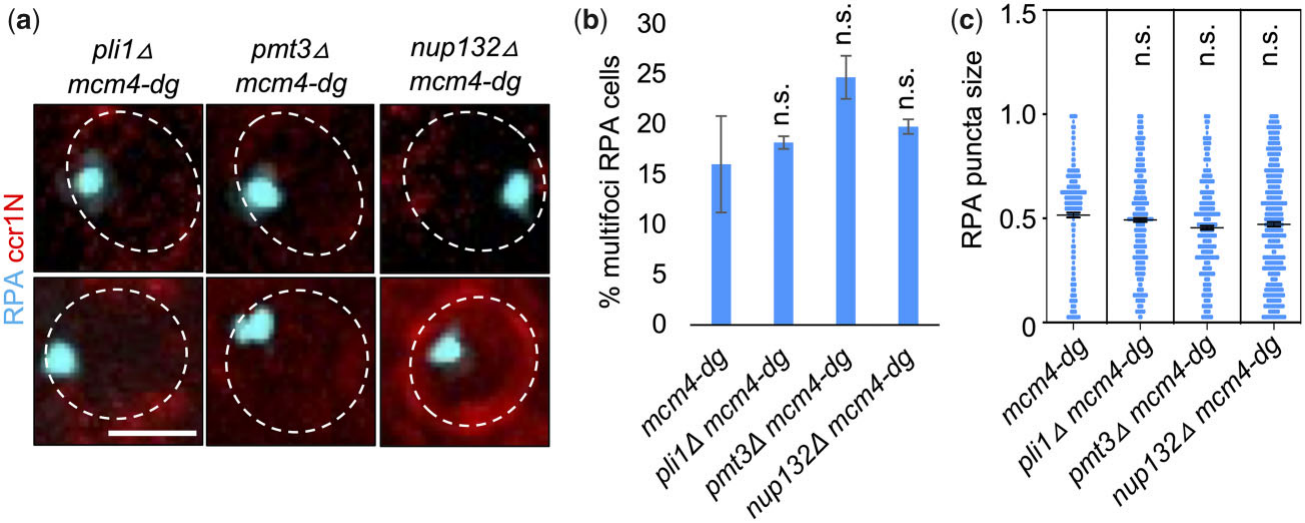
SUMO ligases do not contribute to megafoci RPA formation or its relocalization to nuclear periphery in *mcm4-dg*. a) Nuclei of indicated strains imaged for RPA-CFP and ccr1N-mCherry after 4 h at 36°C (FY9675, FY9676, FY9677). b) Percent of cells containing multifoci RPA from images as in (a). c) Individual RPA puncta size measured using ImageJ from images as in (a) and (b). Scale bar 2 µm. n.s. not significant.

Several pathways have been implicated in the relocalizaton of DNA damage foci to the nuclear periphery. For example, replication forks blocked at a replication fork barrier (RFB) are relocated to the nuclear periphery after poly-SUMOylation by the E3 SUMO ligase Pli1 (Kramarz *et al.* 2020). However, we found that loss of the SUMO ligase Pli1, SUMO gene *pmt3*, or Nup132, a component of the NPC that anchors the SUMOylated replication forks in *mcm4-dg*, did not affect megafoci RPA formation or its relocalization to nuclear periphery (Fig. 5, a–c). Thus, the replication fork structure in *mcm4-dg* do not depend on Pli1-mediated SUMO chains to relocate to nuclear periphery.

**Fig. 6.**
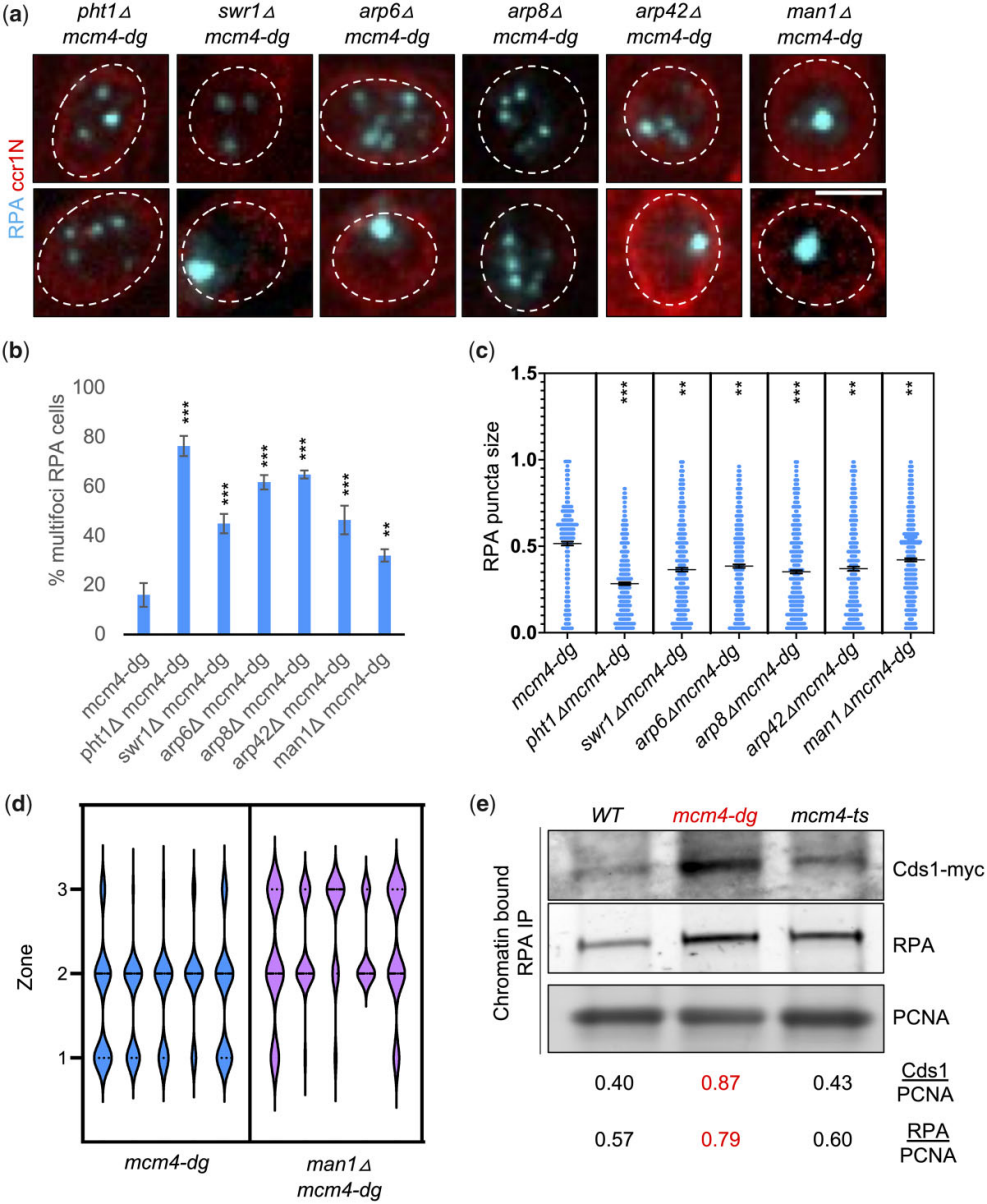
Swr1/Ino80 pathway is required for RPA relocalization to nuclear periphery in *mcm4-dg*. a) Nuclei of indicated strains imaged for RPA-CFP and ccr1N-mCherry after 4 h at 36°C (FY9691, FY9690, FY9725, FY9689, FY9688, FY9693). Scale bar 2 µm. b) Percent of cells containing multifoci RPA from images as in (a). c) Individual RPA puncta size measured using ImageJ from images as in (a). d) Distribution of megafoci RPA over 4 h at 36°C in different zones as defined in Fig. 1b. Five replicates shown. e) Chromatin-bound fractions from *WT*, *mcm4-dg*, *mcm4-ts* lysates were immunoprecipitated against RPA and probed for Cds1-myc (FY10125, FY10126, FY10127). Bottom: band intensity quantified using ImageJ and normalized to PCNA. **P < 0.01, ***P < 0.001.

In budding yeast, the chromatin remodelers Swr1 and Ino80, and Swr1-dependent incorporation of histone H2A variant H2A.Z are required for in the translocation of DSB to Mps3, an INM SUN domain protein (Horigome *et al.* 2014). We tested the contribution of these components in our fission yeast system. We find that loss of Pht1 (the histone H2AZ variant), components of Swr1 complex (Swr1, Arp6), or components of Ino80 complex (Arp8, Arp42) all increase multifoci RPA in *mcm4-dg* background, and these foci remain in the nuclear center (Fig. 6, a–c). Occasionally (10–30%), some cells show a megafocus RPA which is able to locate to the nuclear periphery. Cells deficient in Man1, an LEM domain nuclear inner membrane protein that interacts with Sad1 (ortholog of Mps3; Miki *et al.* 2004; Hiraoka *et al.* 2011), were able to form megafoci RPA with only slightly increased multifoci RPA (Fig. 6, a–c). However, most of the megafoci RPA in *man1Δ mcm4-dg* failed to associate with the nuclear periphery, staying much more in zone 3 (center of nucleus) compared to *mcm4-dg* RPA (Fig. 6d). This suggests that the pathway identified by Horigome *et al.* (2014) is linked to the relocalization we observe, with a role for the Swr1 and Ino80 complex, and Man1 providing a peripheral anchor. Perturbation in Swr1/Ino80/Man1 pathway did not uniformly affect *mcm4-dg* viability (Supplementary Fig. 3a). Lack of Ino80 complex (Arp8 and Arp42) increased *mcm4-dg* viability but neither loss of Swr1 complex (Swr1 and Arp6) nor Man1 had any significant effect. In addition, Pht1 deficiency slightly decreased *mcm4-dg* viability. This suggests that megafocus RPA at nuclear periphery may indicate attempts at repair but fail to have correlation with viability, likely due to multifaceted functions of Swr1/Ino80 as chromatin remodelers (Poli *et al.* 2017).

We also tested a subset of histone modifiers associated with acetylation or methylation including *rtt109Δ* (Xhemalce *et al.* 2007), *mst2Δ* (Reddy *et al.* 2011), *gcn5Δ* (Helmlinger *et al.* 2008), *clr3Δ* (Bjerling *et al.* 2002), *set1Δ* (Noma and Grewal 2002), and *clr4Δ* (Rea *et al.* 2000). None of these affected megafocus formation or its localization at the nuclear periphery in *mcm4-dg* (Supplementary Fig. 3b).

### Increased recruitment of Cds1 to RPA foci leads to aberrant cell cycle

Replication stress in mammalian cells have been shown to amplify replication checkpoint responses by increasing local crowding of ATR to accumulated RPA at stressed replication forks (Yin *et al.* 2021). Coalescence of multifoci RPA into a single large foci (Fig. 1a; Kim and Forsburg 2020) and the recruitment of repair proteins to RPA (Fig. 2) is reminiscent of this RPA-ATR crowding observed in mammalian cells. We showed previously that *mcm4-dg* cells have downregulation of Mus81 by activated Cds1 that leads to evasion of Chk1 (Kim and Forsburg 2020), so we hypothesized that *mcm4-dg* increase recruitment of Cds1 to stressed replication forks via interaction with RPA. To test this, we immunoprecipitate RPA from *mcm4-dg* lysates prepared after 4 h at the restrictive temperature and probed for Cds1. WT and the canonical temperature-sensitive *mcm4-ts* that lack megafoci RPA and Cds1 hyperactivity (Sabatinos *et al.* 2015; Kim and Forsburg 2020) were included for comparison. All three strains contained Cds1 in both chromatin-bound insoluble fraction and chromatin-unbound soluble fraction (Fig. 6e and Supplementary Fig. 4). However, *mcm4-dg* contained more Cds1 that interacted with RPA in insoluble fraction compared to WT or *mcm4-ts*. These results suggest that megafoci RPA in *mcm4-dg* results in enhanced Cds1 recruitment to chromatin that correlates to hyperactivity of Cds1.

### Cell cycle dynamics in *mcm4-dg*

The formation of the megafocus in *mcm4-dg* is accompanied by several rounds of cell division even at the restrictive temperature (Sabatinos *et al.* 2015). One possibility is that cells are undergoing an extra cell cycle despite the failure in DNA replication. We examined the dynamics of megafocus formation relative to cell cycle markers in these cells to assess the dynamics of the cell division process.

**Fig. 7.**
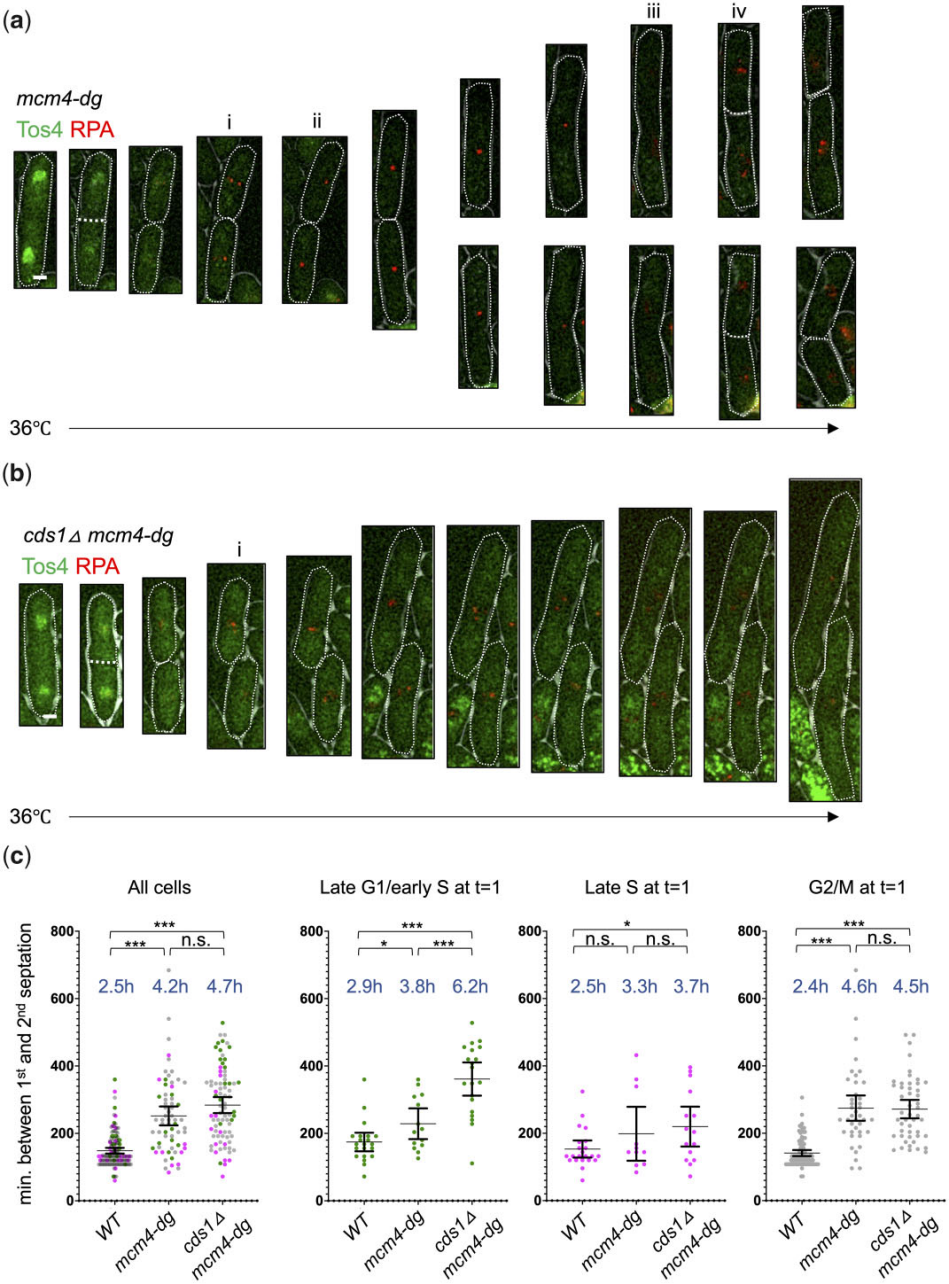
Replication stress in early S phase requires Cds1 to divide in timely manner. a, b) Time-lapse images of *mcm4-dg* or *cds1Δ mcm4-dg* cells for Tos4-GFP and RPA-mCherry at 36°C (FY10089, FY10098). (i) Initial appearance of RPA. (ii) Coalesced RPA. (iii) Disappearance of RPA before septation. (iv) Reappearance of RPA near septation. Scale bar 2 µm. c) Minutes between first and second septation from time-lapse images of *mcm4-dg* or *cds1Δmcm4-dg* cells. Late G1/early S phase indicated by binucleate Tos4-GFP. Late S phase indicated by mononucleate Tos4-GFP. G2/M phase indicated by lack of nuclear Tos4-GFP. *P < 0.05, ***P < 0.001, n.s.: not significant.

**Fig. 8.**
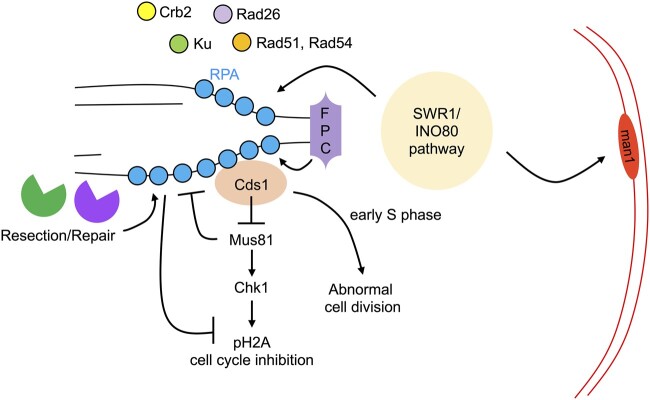
Replication stress in *mcm4-dg* that allows evasion of checkpoint has distinct RPA foci phenotype. Resection and repair machinery and FPC contribute to megafoci RPA formation. Swr1/Ino80 pathway also contributes to megafoci formation and is required to bring megafoci RPA to the nuclear periphery. Repair proteins like Ku70, Crb2, Rad51, Rad54, and Rad26 are recruited to RPA foci. Cds1 contributes to cell division especially in cells that are depleted of Mcm4 during early S phase.

Tos4 is a FHA domain protein that undergoes transcription and nuclear localization during G1/S phase transition and is a useful tool in assessing cell cycle through S phase (Bastos de Oliveira *et al.* 2012; Kim *et al.* 2020). We used GFP-tagged Tos4 to follow cells that passed into S phase at the start of incubation at the restrictive temperature. In fission yeast, S phase occurs in binucleate cells just before septation (Gomez and Forsburg 2004). In agreement with our previous work, multifoci RPA in *mcm4-dg* appeared soon after nuclear Tos4 disappeared at septation (Kim and Forsburg 2020) (Fig. 7a, i). Multifoci RPA coalesced into a single foci and then disappeared as next division occurred (Fig. 8a, ii, iii). Multifoci RPA appeared again with the second septation (Fig. 7a, iv). Interestingly, although cells septated, nuclear Tos4 did not reappear in the during the second division (Fig. 7a, iii–iv). WT cells in the same conditions showed nuclear Tos4 in multiple sequential cell divisions (Supplementary Fig. 5). Despite the absence of Tos4 incorporation, we observed transient formation of a mitotic spindle before each septation suggesting that *mcm4-dg* cells do undergo M phase properly (Supplementary Fig. 6). However, it is possible that they are bypassing S phase.

Checkpoint kinases regulate gene expression during G1/S, including Tos4 (reviewed in Bertoli *et al.* 2013). Our previous data showed that *mcm4-dg* phenotypes rely on Cds1; in the absence of Cds1, cells have a high fraction of multifoci and do not show the same aberrant divisions (Kim and Forsburg 2020). We performed timelapse imaging of Tos4-GFP and RPA in *cds1Δ mcm4-dg* cells passing through S phase. As with *mcm4-dg*, RPA foci appeared after nuclear Tos4 disappeared and septation occurred (Fig. 7b, i). However, *cds1Δ mcm4-dg* cells elongated instead of entering second division, consistent with a delay in cell cycle. For those cells that did divide twice, we measured the time between first and second septation. Compared to WT cells, *mcm4-dg* cells overall had longer period between septations (Fig. 7c). The cell cycle delay observed in *cds1Δ mcm4-dg* was specific to cells that were placed at restrictive temperature while in G1/S phase (binucleate cells that have nuclear Tos4 at the start of imaging) (Fig. 7c). Compared to *mcm4-dg* G1/S cells that take less than 4 h to complete cell cycle, *cds1Δ mcm4-dg* cells took longer than 6 h. Cells that were at late S (mononucleate cells with nuclear Tos4) or G2/M (mononucleate cells without nuclear Tos4) were not affected by Cds1 deficiency. Although Cds1 deletion in *mcm4-dg* delayed cell cycle in G1/S cells, greater percentage of cells were able to undergo cell division eventually (Supplementary Fig. 7), confirming that Cds1 contributes to toxicity *mcm4-dg* cells encounter.

## Discussion

The response to DNA replication stress depends on management of ssDNA (rev in Sabatinos and Forsburg 2015; Cortez 2019). Formation of ssDNA in replication stress can result from several processes, including resection, helicase/replisome uncoupling, formation of ssDNA gaps due to lesion bypass, and D-loop formation in recombination. The ssDNA-binding protein RPA is a monitor for ssDNA accumulation and acts as a limiting factor for managing replication stress (Toledo *et al.* 2017; Bhat and Cortez 2018; Ercilla *et al.* 2020). We have previously used patterns of RPA in live fission yeast cells to help distinguish different forms of replication stress (Sabatinos *et al.* 2012, 2015).

In this report, we characterize the formation and dynamics of large RPA megafoci in *S. pombe mcm4-dg* mutants at the restrictive temperature. Unlike other *mcm4* temperature-sensitive alleles, the *mcm4-dg* strain has a profound defect in DNA replication but is unable to undergo checkpoint mediated arrest (Lindner *et al.* 2002; Bailis *et al.* 2008; Sabatinos *et al.* 2015). The *mcm4-*dg megafoci are distinct from multiple dispersed puncta of RPA (multifoci) observed in other *mcm4* temperature-sensitive mutants (Sabatinos *et al.* 2015; Ranatunga and Forsburg 2016). We previously showed that megafocus formation occurs by the merging of multifoci and relocalization to the nuclear periphery, in a pathway that depends on the Cds1-mediated repression of Mus81 endonuclease, and that also depends on the end-binding protein Ku (Kim and Forsburg 2020). These observations suggest that processing of distinct fork structures in *mcm4-dg* is important for the morphology and localization of the RPA megafoci. In this report, we further characterize megafocus formation and investigated the contribution of repair and remodeling factors to this structure.

We observed that the Rad3 (ATR) activating protein Rad26 (ATRIP), which is known to bind RPA (Yin *et al.* 2021), is recruited efficiently to the megafocus. Crb2 (53BP1), another checkpoint mediator, is also observed at both multi- and mega-foci. Previously, we showed that the recombination mediator Rad52 is part of the megafocus (Sabatinos *et al.* 2015) and, consistent with this, we also saw recruitment of Rad51 and Rad54. This was not a surprise, since numerous studies suggest that HR proteins, particularly Rad51, play a key role in replication fork stress response (Bhat and Cortez 2018; Nickoloff *et al.* 2021; Spies *et al.* 2021). However, the GCR and chromosome loss associated with *mcm4-dg* is not significantly affected by the loss of HR proteins (Fig. 2e), indicating that this is not an HR-dependent outcome.

The Ku protein complex has a key role in the formation of megafocus RPA (Kim and Forsburg 2020). Ku is an DNA end-binding protein associated with non-homologous end joining (Zahid *et al.* 2021) but is also linked to the recognition of “one-sided” DNA ends and replication fork regression (Foster *et al.* 2011; Langerak *et al.* 2011; Teixeira-Silva *et al.* 2017). Loss of Ku70 in an *mcm4-dg* background prevents formation of megafoci (Kim and Forsburg 2020) and also prevents recruitment of the Crb2 protein (Fig. 2). In addition to its role in checkpoint activation by binding DNA ends (Sofueva *et al.* 2010; Qu *et al.* 2012), Crb2 opposed excessive break resection (Leland *et al.* 2018). We suggest that the Ku70 foci in the megafocus reflect formation of single-end DSBs (seDSBs). Ku removal from seDSB ends by MRN is stimulated by Ctp1 direct repair to HR (Langerak *et al.* 2011) and Rad51-mediated fork reversal and recombination opposes the formation of seDSBs (Whelan *et al.* 2020). seDSBs can be repaired by break-induced replication (BIR), a subset of HR, which involves Rad51 nucleoprotein filament invasion of sister chromatid (Kramara *et al.* 2018; Nickoloff *et al.* 2021). Presence of both Ku and Rad51 in *mcm4-dg* RPA foci (Fig. 2) may reflect a mixture of processed and unprocessed seDSBs.

Recruitment of repair proteins to RPA foci suggests that there is an attempt to resolve failed replication structures or subsequent DNA lesions. Recruitment of Rad26, homolog of ATRIP, to RPA and increased RPA-Cds1 interaction in *mcm4-dg* suggest that localized crowding of RPA-Cds1 may be responsible for hyperactivity of Cds1 in *mcm4-dg*. This is similar to ATR-CHK1 signal propagation at replication forks via RPA-ATR interaction observed in mammalian cells (Yin *et al.* 2021). Decreased Cds1 activation likely contributes to increased multifoci RPA formation in Rad26-deficient *mcm4-dg* cells (Supplementary Fig. 1d). As Mrc1 mediates Rad3-Rad26 activation of Cds1 (Tanaka and Russell 2004), it is possible that Chk1 remains inactivated while Cds1 is activated in *mcm4-dg* through mechanisms involving Mrc1 and other components of the FPC. These findings reveal a mechanism by which aberrant hyperactivation of the replication checkpoint can take place and contribute to genome instability.

Since management of ssDNA appears to be critical for the formation of the megafocus, we examined contribution of known remodelers. We observed that megafocus formation was significantly reduced in mutant that affect resection (*exo1*, *rqh1*; Willis and Rhind 2009; Saada *et al.* 2017), flap processing (*fan1*) or mutants that result in replication fork uncoupling (FPC, *swi1*, *swi3*, *mrc1*). Our previous work showed that the activation of endonuclease Mus81 also prevents megafocus formation. We conclude that megafocus formation depends on resection. Why megafoci RPA in *mcm4-dg* require extensive resection may be tied to the types of potential replication intermediates generated in *mcm4-dg*. A recent study demonstrated that failure to limit seDSBs in Abraxas/BRCA1-A complex deficient mammalian cells leads to excessive resection and increased BIR (Wu and Wang 2021). It is feasible that unprocessed seDSBs in *mcm4-dg* result in extensive resection, which result in increased RPA binding. Why putative seDSBs in *mcm4-dg* are not efficiently repaired despite the presence of repair proteins requires further investigation.

We next sought to identify other pathways involved in formation and localization of the megafocus to the periphery. We found that loss of SUMO proteins or Nup132 *mcm4-dg* did not affect megafoci RPA formation or its localization to the nuclear periphery (Fig. 5). Thus, replication stress response induced by the loss of Mcm4 protein in *mcm4-dg* appears to be distinct from that induced by the RFB or heterochromatin breaks (Ryu *et al.* 2015; Kramarz *et al.* 2020). This would be consistent with the absence of obvious DSB signals including phospho-H2A(X) in *mcm4-dg* (Bailis *et al.* 2008). However, we cannot rule out that other sources of SUMOylation contribute to the RPA phenotype observed in *mcm4-dg*. For instance, in human cells, SLX4, the interactor of ERCC4-ERCC1, MUS81-EME1, and SLX1 endonucleases, acts as a SUMO E3 ligase and its SUMOylation activity increases toxicity in cells with global replication fork stalling (Guervilly *et al.* 2015). It is feasible that these types of other SUMO ligases are involved in SUMOylation of components of replication structures in *mcm4-dg* that contribute to its RPA phenotype and relocalization to nuclear periphery.

In contrast, Swr1/Ino80 chromatin remodelers and the histone variant H2A.Z (Pht12) play a role in the relocalization of RPA foci to the nuclear periphery and nuclear inner membrane protein Man1 helps anchor it, similar to the relocalization pathway identified in (Horigome *et al.* 2014).

Finally, we examined the cell cycle dynamics of *mcm4-dg*. Consistent with the roles of MCM helicase in DNA synthesis, Cds1 loss delayed cell cycle progression in cells that lost Mcm4 in early S phase but not in cells that lost Mcm4 in G2 or M phase (Fig. 8). This is also consistent with our previous finding that hyperactive Cds1 contributes to evasion of cell cycle arrest in replication-stressed *mcm4-dg* (Kim and Forsburg 2020). However, we find that hyperactive Cds1 in *mcm4-dg* does not induce expression of Tos4, which is normally upregulated by the Mlu1-binding factor (MBF)-mediated G1/S transcription and Cds1 downregulation of the transcription corepressors Nrm1 and Yox1 (rev in Bertoli *et al.* 2013). It is possible that Cds1 loses activity during second and subsequent cycles. Or it may indicate that Cds1 recruited to RPA foci does not inactivate Nrm1 and Yox1 or that other aberrant replication stress signals or lack of DNA synthesis prevents MBF-mediated transcription. Either way, this would blur the distinction between S phase and G2 phase cells.

This study demonstrates a unique type of replication stress observed in Mcm4 helicase mutant *mcm4-dg* that results in a distinctive RPA foci pattern and evasion of cell cycle arrest. Our findings add to targets of DNA repair taking place at the nuclear periphery and highlight the importance of links between replication stress response and abnormal cell division.

## Data availability

Strains and plasmids are available upon request. Supplemental files including strain table and supplemental figures are available at G3 online.

Supplemental material is available at *G3* online.

## Supplementary Material

jkac116_Supplementary_Data
