# Supplementary material for: Determinants of RPA megafoci localization to the nuclear periphery in response to replication stress
Source: G3 (Bethesda). 2022 May 14;12(7):jkac116. doi: 10.1093/g3journal/jkac116 (PMC9258583; doi:10.1093/g3journal/jkac116)
Supplement: jkac116_Supplementary_Data [file jkac116_supplementary_data.pdf]

Strain #	Genotype	Source	Reference
FY4003	h- ChL[ubcp4::LEU2::chk1 kan:spccB3.18 spcc1322.09::ura4+ ade6+] ade6Δ-D ura4-D18 leu1-32	Takuro Nakagawa	Nakamura et al. 2008
FY7423	h+ mcm4(cdc21-M68)-ts-dg::ura4+ arg3+::ccr1N-mCherry(D817 aa1-275)::his5+ rad11-CFP-hphMX rad22-YFP-NatMX ura4-D18	Our stock	
FY7426	h- arg3+::ccr1N-mCherry(D817 aa1-275)::his5+ rad11-CFP-hphMX rad22-YFP-NatMX ura4-D18 leu1-32	Our stock	
FY8770	h+ cdc21-ts-dg::ura4+ (mcm4) Δexo1::ura4+ rad22-YFP::natMX6 rad11-CFP::hphMX6 ura4-D18	Our stock	
FY9405	h+ mcm4(cdc21-M68)-ts-dg::ura4+ arg3+::ccr1N-mCherry(D817 aa1-275)::his5+ pKu70-citrinehph rad11-Cerulean::hphMX ura4-D18	Our stock	
FY9431	h- cdc21-M68-ts-dg::ura4+ ChL[ubcp4::LEU2::chk1 hph:spccB3.18 spcc1322.09::ura4+ ade6+] ade6-Δ ura4-D18 leu1-32 ade6-M210	Takuro Nakagawa	Nakamura et al. 2008
FY9447	h+ mcm4(cdc21-M68)-ts-dg::ura4+ nup107-tomato::natMX4 rad11-Cerulean::hphMX leu1-32 ura4-D18	Our stock	
FY9639	h- mcm4(cdc21-M68)-ts-dg::ura4+ leu1-32::[2x YFP-crb2-leu1+] nup107-tomato::natMX4 rad11-Cerulean::hphMX ura4-D18	Steven L. Sanders	Sanders et al. 2004
FY9675	h90 Δpli1::kanMX mcm4(cdc21-M68)-ts-dg::ura4+ arg3+::ccr1N-mCherry(D817 aa1-275)::his5+ rad11-CFP-hphMX rad22-YFP-NatMX ura4-D18	This study	
FY9676	h+ Δpmt3::kanMX mcm4(cdc21-M68)-ts-dg::ura4+ arg3+::ccr1N-mCherry(D817 aa1-275)::his5+ rad11-CFP-hphMX rad22-YFP-NatMX ura4-D18	This study	
FY9677	h+ Δnup132::kanMX mcm4(cdc21-M68)-ts-dg::ura4+ arg3+::ccr1N-mCherry(D817 aa1-275)::his5+ rad11-CFP-hphMX rad22-YFP-NatMX ura4-D18	This study	
FY9688	h+ Δarp42::kanMX4 mcm4(cdc21-M68)-ts-dg::ura4+ arg3+::ccr1N-mCherry(D817 aa1-275)::his5+ rad11-CFP-hphMX rad22-YFP-NatMX ura4-D18	Our stock	
FY9689	h+ Δarp8::kanMX4 mcm4(cdc21-M68)-ts-dg::ura4+ arg3+::ccr1N-mCherry(D817 aa1-275)::his5+ rad11-CFP-hphMX rad22-YFP-NatMX ura4-D18	Our stock	
FY9690	h+ Δswr1::kanMX6 mcm4(cdc21-M68)-ts-dg::ura4+ arg3+::ccr1N-mCherry(D817 aa1-275)::his5+ rad11-CFP-hphMX rad22-YFP-NatMX ura4-D18	Our stock	
FY9691	h90 pht1::ura4 mcm4(cdc21-M68)-ts-dg::ura4+ arg3+::ccr1N-mCherry(D817 aa1-275)::his5+ rad11-Cerulean::hphMX rad22-YFP-natMX ura4-D18	Nancy Walworth (originally from Antony Carr)	Ahmed et al. 2007
FY9693	h90 Δman1::kanMX mcm4(cdc21-M68)-ts-dg::ura4+ arg3+::ccr1N-mCherry(D817 aa1-275)::his5+ rad11-Cerulean::hphMX rad22-YFP-natMX ura4-D18	Shelley Sazer	Gonzalez et al. 2012
FY9705	h- Δrqh1:kanMX6 mcm4(cdc21-M68)-ts-dg::ura4+ arg3+::ccr1N-mCherry(D817 aa1-275)::his5+ rad11-CFP-hphMX ura4-D18	Our stock	
FY9725	h- arp6::ura4+ mcm4(cdc21-M68)-ts-dg::ura4+ arg3+::ccr1N-mCherry(D817 aa1-275)::his5+ rad11-CFP-hphMX rad22-YFP-NatMX ura4-D18	Masahiko Harata	Ueno et al. 2004
FY9884	h+ fan1Δ::kan mcm4(cdc21-M68)-ts-dg::ura4+ arg3+::ccr1N-mCherry(D817 aa1-275)::his5+ rad11-CFP-hphMX rad22-YFP-NatMX ura4-D18	Our stock	
FY9894	h+ cdc21-M68-ts-dg::ura4+ Ssb2-mCherry-NatR rad26 GFP:: Kan MX ura4-D18 or ura4-294 leu1-32	Tom Wolkow	Wolkow and Enoch 2003
FY9926	h+ Δfbh1::kanMX mcm4(cdc21-M68)-ts-dg::ura4+ arg3+::ccr1N-mCherry(D817 aa1-275)::his5+ rad11-CFP-hphMX rad22-YFP-NatMX ura4-D18	Our stock	
FY9939	h- Δmrc1::kanMX6 mcm4(cdc21-M68)-ts-dg::ura4+ rad11-CFP-hphMX rad22-YFP-NatMX ura4-D18	Our stock	
FY9945	h- cdc21-M68-ts-dg::ura4 rad54-GFP:hphMX6 Ssb2-mCherry-NatR leu1-32 ura4-D18	Takuro Nakagawa	Maki et al. 2011
FY9951	h- Δmrc1::ura4+ leu1+::(mrc1(T645A + T653A)-3HA) mcm4(cdc21-M68)-ts-dg::ura4+ arg3+::ccr1N-mCherry(D817 aa1-275)::his5+ rad11-CFP-hphMX rad22-YFP-NatMX ura4-D18 leu1-32	Our stock	
FY9987	h+ Δswi1::kanMX6 mcm4(cdc21-M68)-ts-dg::ura4+ arg3+::ccr1N-mCherry(D817 aa1-275)::his5+ rad11-CFP-hphMX rad22-YFP-NatMX ura4-D18 leu1-32	Paul Russell	Noguchi et al. 2004
FY9989	h+ Δswi3::KanMX6 mcm4(cdc21-M68)-ts-dg::ura4+ arg3+::ccr1N-mCherry(D817 aa1-275)::his5+ rad11-CFP-hphMX rad22-YFP-NatMX ura4-D18 leu1-32	Paul Russell	Noguchi et al. 2004
FY9991	h+ Δrad26::ura4+ mcm4(cdc21-M68)-ts-dg::ura4+ arg3+::ccr1N-mCherry(D817 aa1-275)::his5+ rad11-CFP-hphMX rad22-YFP-NatMX ura4-D18	Tom Wolkow	Wolkow and Enoch 2003
FY10003	h- pku70::kanr cdc21-M68-ts-dg::ura4 Δcrb2::ura4+ leu1-32::[leu1+ pJK148-REP81-GFP-crb2] Ssb2-mCherry-NatR leu1-32 ura4-D18	Our stock	
FY10005	h- Δrad26::ura4+ cdc21-M68-ts-dg::ura4 Δcrb2::ura4+ leu1-32::[leu1+ pJK148-REP81-GFP-crb2] Ssb2-mCherry-NatR leu1-32 ura4-D18	Our stock	
FY10006	h+ pku70::kanr cdc21-M68-ts-dg::ura4+ Ssb2-mCherry-NatR rad26 GFP:: Kan MX ura4-D18 or ura4-294 leu1-32	Our stock	
FY10007	h+ rhp54Δ::ura4+ cdc21-M68-ts-dg::ura4+ Ssb2-mCherry-NatR rad26-GFP:: Kan MX ura4-D18 or ura4-294 leu1-32	Our stock	
FY10008	h+ Δrhp9::ura4+ cdc21-M68-ts-dg::ura4+ Ssb2-mCherry-NatR rad26 GFP:: KanMX ura4-D18 or ura4-294 leu1-32	Our stock	
FY10009	h- crb2::kanMX6 mcm4(cdc21-M68)-ts-dg::ura4+ arg3+::ccr1N-mCherry(D817 aa1-275)::his5+ rad11-CFP-hphMX rad22-YFP-NatMX ura4-D18	Our stock	
FY10010	h- pku70::kanr cdc21-M68-ts-dg::ura4 rad54-GFP:hphMX6 Ssb2-mCherry-NatR leu1-32 ura4-D18	Our stock	
FY10011	h+ crb2::kanMX6 cdc21-M68-ts-dg::ura4 rad54-GFP:hphMX6 Ssb2-mCherry-NatR leu1-32 ura4-D18	Our stock	
FY10018	h- cdc21-M68-ts-dg::ura4 rhp51-eCFP-kanMX Ssb2-mCherry-NatR leu1-32 (rad51+)	Timothy C. Humphrey	Aronica et al. 2016
FY10021	h- rhp54::kanMX6 cdc21-M68-ts-dg::ura4 Δcrb2::ura4+ leu1-32::[leu1+ pJK148-REP81-GFP-crb2] Ssb2-mCherry-NatR leu1-32 ura4-D18	Our stock	
FY10025	h- rhp54::kanMX6 cdc21-M68-ts-dg::ura4+ ChL[ubcp4::LEU2::chk1 hph:spccB3.18 spcc1322.09::ura4+ ade6+] ade6-Δ ura4-D18 leu1-32	Our stock	
FY10029	h- Δrhp51::kanMX6 cdc21-M68-ts-dg::ura4+ ChL[ubcp4::LEU2::chk1 hph:spccB3.18 spcc1322.09::ura4+ ade6+] ade6-Δ ura4-D18 leu1-32	Our stock	
FY10031	h- Δctp1::kanMX6 cdc21-M68-ts-dg::ura4+ ChL[ubcp4::LEU2::chk1 hph:spccB3.18 spcc1322.09::ura4+ ade6+] ade6-Δ ura4-D18 leu1-32	Our stock	
FY10033	h- pku70::kanr cdc21-M68-ts-dg::ura4+ ChL[ubcp4::LEU2::chk1 hph:spccB3.18 spcc1322.09::ura4+ ade6+] ade6-Δ ura4-D18 leu1-32 ade6-M210	Our stock	
FY10035	h- crb2::kanMX6 cdc21-M68-ts-dg::ura4+ ChL[ubcp4::LEU2::chk1 hph:spccB3.18 spcc1322.09::ura4+ ade6+] ade6-Δ ura4-D18 leu1-32	Our stock	
FY10054	h- crb2::kanMX6 cdc21-M68-ts-dg::ura4 rhp51-eCFP-kanMX Ssb2-mCherry-NatR leu1-32	Our stock	
FY10064	h- Ssb2-mCherry-NatR Tos4-GFP::KanMX6 leu1-32 ura4-D18	Our stock	
FY10073	h- Δrad26::ura4+ mcm4 (cdc21-m68)-ts-dg::ura4+ pKu70-citrinehph Ssb2-mCherry-NatR ura4-D18 leu1-32	Our stock	
FY10083	h- Δmst2::kanMX6 mcm4(cdc21-M68)-ts-dg::ura4+ arg3+::ccr1N-mCherry(D817 aa1-275)::his5+ rad11-CFP-hphMX rad22-YFP-NatMX ura4-D18	Our stock	
FY10084	h90 Δclr3::kanMX6 mcm4(cdc21-M68)-ts-dg::ura4+ arg3+::ccr1N-mCherry(D817 aa1-275)::his5+ rad11-CFP-hphMX rad22-YFP-NatMX ura4-D18	Karl Ekwall	Bjerling et al. 2002
FY10085	h- ΔGCN5::kanMX mcm4(cdc21-M68)-ts-dg::ura4+ arg3+::ccr1N-mCherry(D817 aa1-275)::his5+ rad11-CFP-hphMX rad22-YFP-NatMX ura4-D18	Our stock	
FY10086	h+ clr4::kanMX6 mcm4(cdc21-M68)-ts-dg::ura4+ arg3+::ccr1N-mCherry(D817 aa1-275)::his5+ rad11-CFP-hphMX rad22-YFP-NatMX ura4-D18	Our stock	
FY10087	h90 Δset1::kanMX6 mcm4(cdc21-M68)-ts-dg::ura4+ arg3+::ccr1N-mCherry(D817 aa1-275)::his5+ rad11-CFP-hphMX rad22-YFP-NatMX ura4-D18 leu1-32	Our stock	
FY10089	h- cdc21-M68-ts-dg::ura4 Ssb2-mCherry-NatR Tos4-GFP::KanMX6 leu1-32	Our stock	
FY10092	h- cdc21-M68-ts-dg::ura4 Ssb2-mCherry-NatR kanR<<Pnmt41-mCherry-atb2+ arg3+::ccr1N-GFP(D817 aa1-275))::his5+ leu1-32 ura4-D18	Our stock	
FY10098	h- cds1::ura4+ cdc21-M68-ts-dg::ura4 Ssb2-mCherry-NatR Tos4GFP::KanMX6 ura4-D18 leu1-32	Our stock	
FY10125	h+ cds1-myc::KanMX rad11-Cerulean::hphMX ade6-M216 or M210 ura4-D18 leu1-32	Our stock	
FY10126	h+ cdc21-M68 cds1-myc::KanMX rad11-Cerulean::hphMX ade6-M216 or M210 ura4-D18 leu1-32	Our stock	
FY10127	h- mcm4(cdc21-M68)-ts-dg::ura4+ cds1-myc::KanMX rad11-Cerulean::hphMX ura4-D18 leu1-32	Our stock	
FY10145	h+ Δrtt109::kanMX4 mcm4(cdc21-M68)-ts-dg::ura4+ arg3+::ccr1N-mCherry(D817 aa1-275)::his5+ rad11-CFP-hphMX rad22-YFP-NatMX ura4-D18	Our stock	

Supplementary Table 1. List of strains

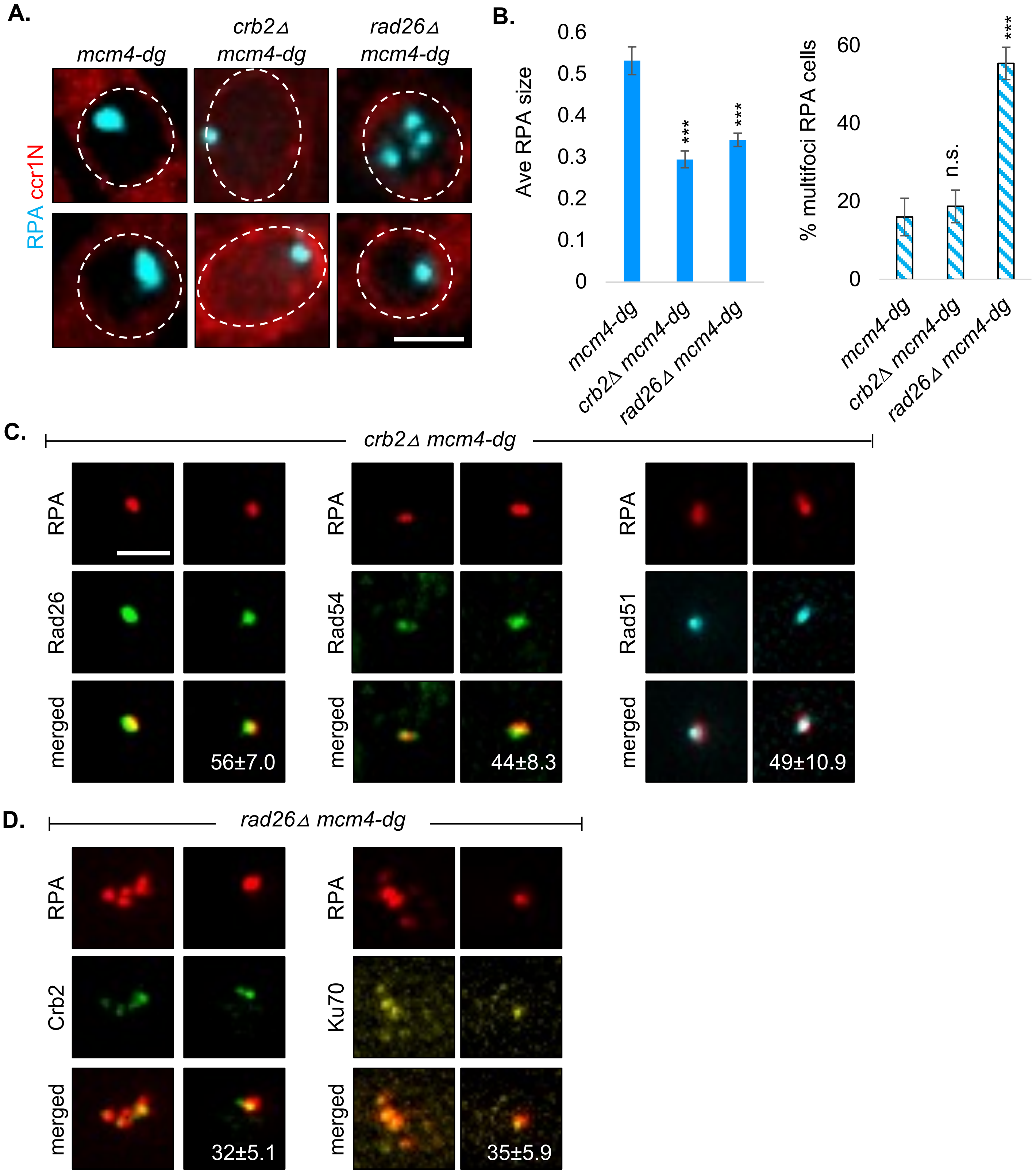

Supplementary Figure 1. Crb2 or Rad26 deficiency affects RPA phenotype in *mcm4-dg* but does not affect repair protein recruitment. (A) Nuclei of indicated strains imaged for RPA-CFP and ccr1N-mCherry after 4 h at 36℃ (FY7423, FY10009, FY9991). (B) Average RPA foci size and percent of cells containing multifoci RPA from images as in (A). (C-D) Nuclei of indicated strains imaged for RPA-mCherry with Rad26-GFP, Rad54-GFP, Rad51-CFP, Crb2-GFP, or Ku70-YFP, after 4 h at 36℃ (FY10008, FY10011, FY10054, FY10005, FY10073). Numbers on the bottom images indicate % RPA that colocalizes with the repair protein (mean ± S.E.). Scale bar 2 μm.

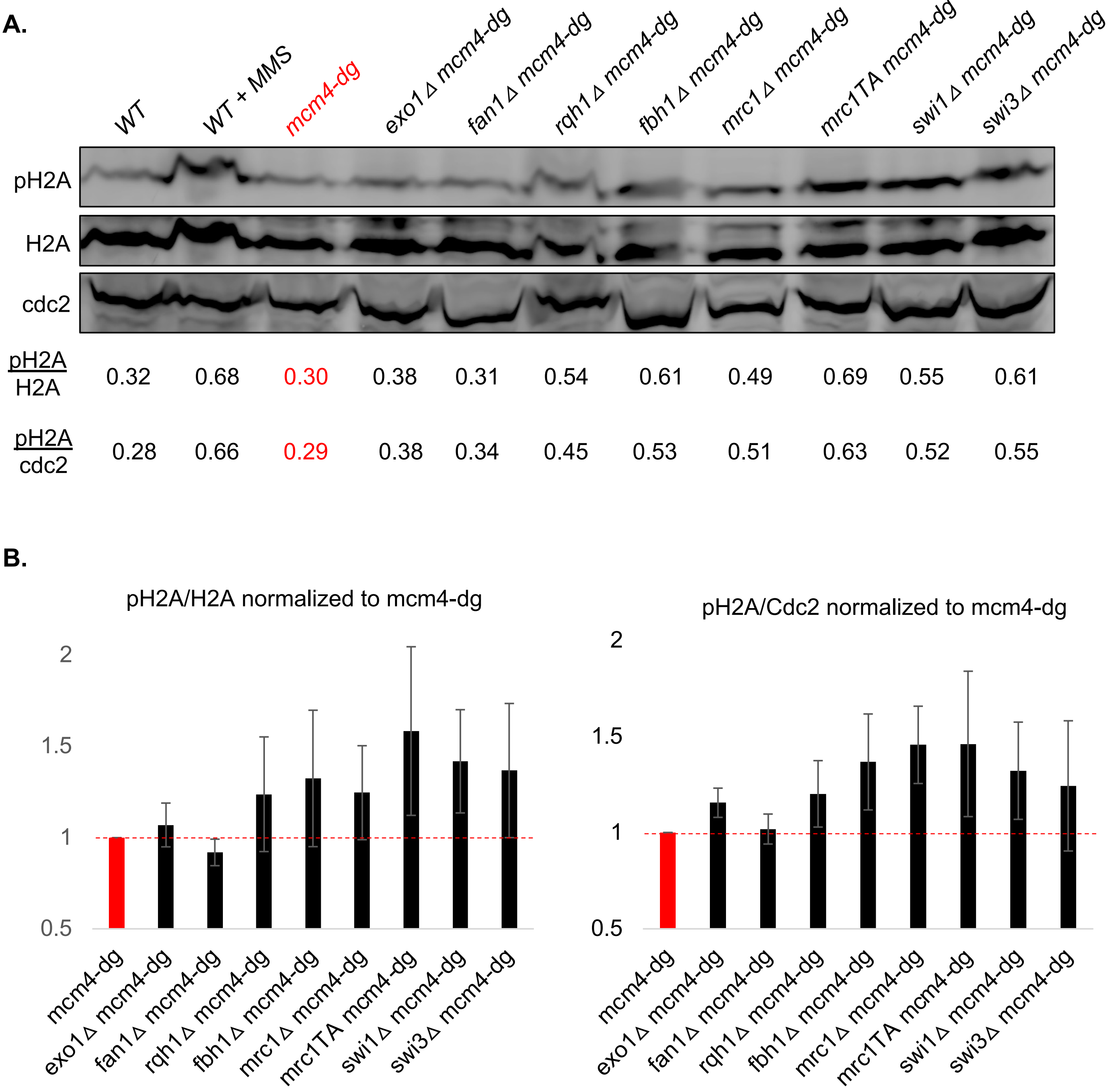

Supplementary Figure 2. Defects in megafoci RPA formation (increase in multifoci RPA) is correlated with increased phosphorylation of H2A. (A) Western blot for phospho-H2A, H2A, and cdc2 loading control (FY7426, FY7423, FY8770, FY9884, FY9705, FY9926, FY9939, FY9951, FY9987, FY9989). 0.007% MMS-treated WT included as positive control. Quantification of band intensity ratio is shown below. (B) Quantification of band intensity ratio normalized to *mcm4-dg* from 3 biological replicates.

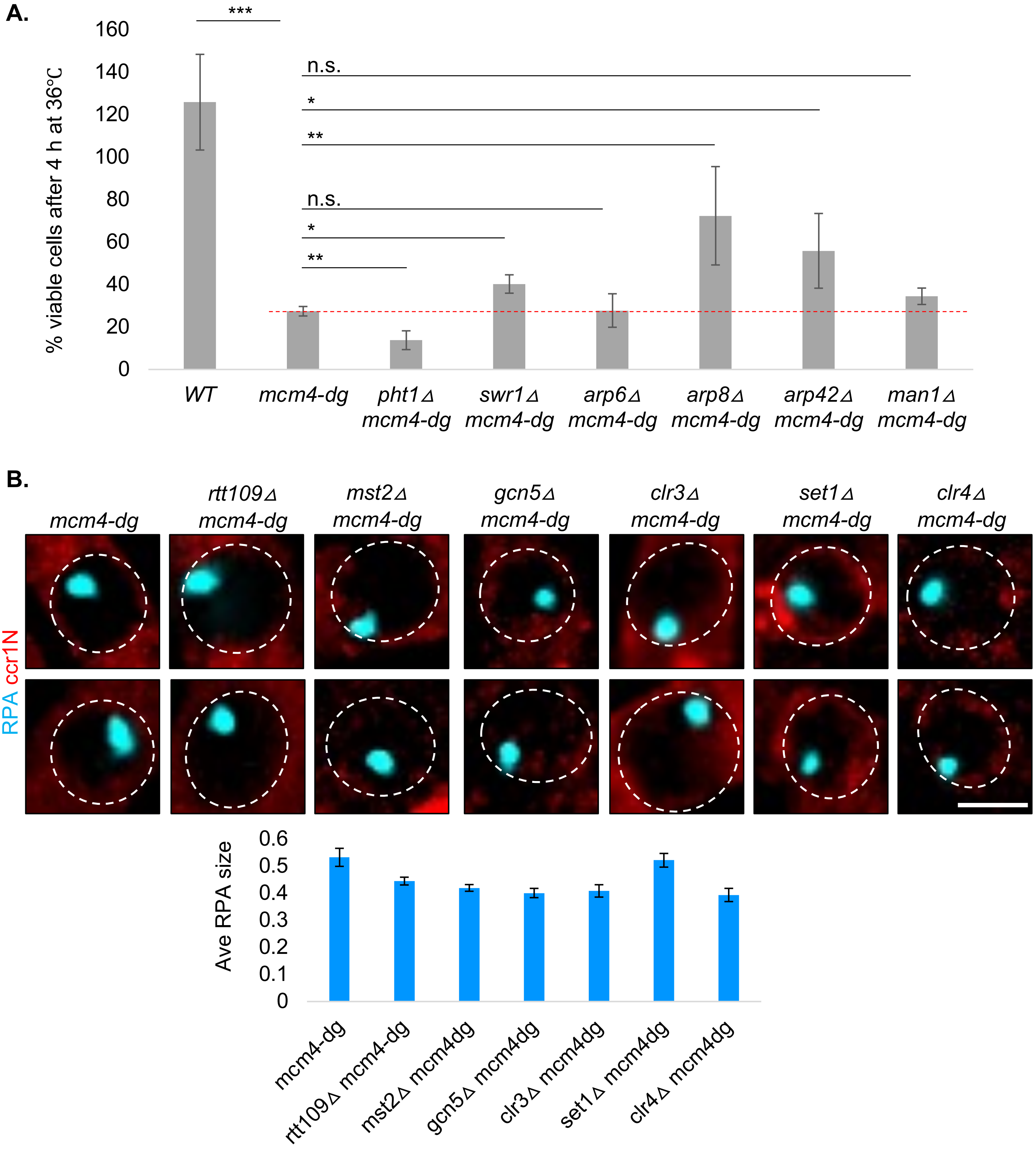

Supplementary Figure 3. Swr1/Ino80 pathway does not uniformly affect viability in mcm4-dg and histone acetylation or methylation do not affect megafoci RPA formation in *mcm4-dg*. (A) Indicated strains were placed at 36℃ for 4 h and same number of cells were plated on YES plate and incubated at 25℃ (FY7426, FY7423, FY9691, FY9690, FY9725, FY9689, FY9688, FY9693). (B) Nuclei of indicated strains imaged for RPA-CFP and ccr1N-mCherry after 4 h at 36℃ (FY7423, FY10145, FY10083, FY10084, FY10085, FY10086, FY10087). Bottom, percent of cells containing multifoci RPA from images as in (A). Scale bar 2 μm.

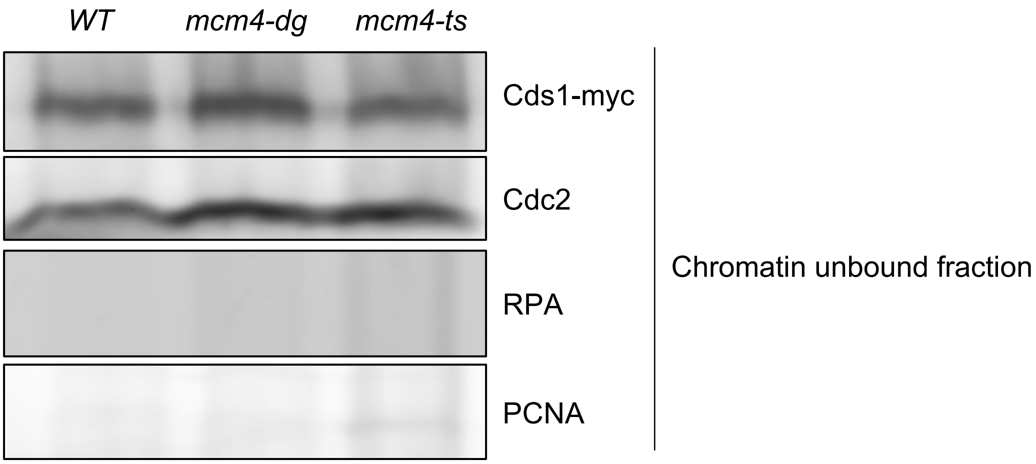

Supplementary Figure 4. Swr1/Ino80 pathway does not uniformly affect viability in *mcm4-dg* and chromatin unbound fraction in mcm4 mutants contain Cds1. After 4 h at 36℃, indicated strains were lysed and chromatin bound and unbound fractions were separated by centrifugation (FY10125, FY10126, FY10127). Chromatin unbound fraction lacked RPA and PCNA but contained Cds1 and Cdc2.

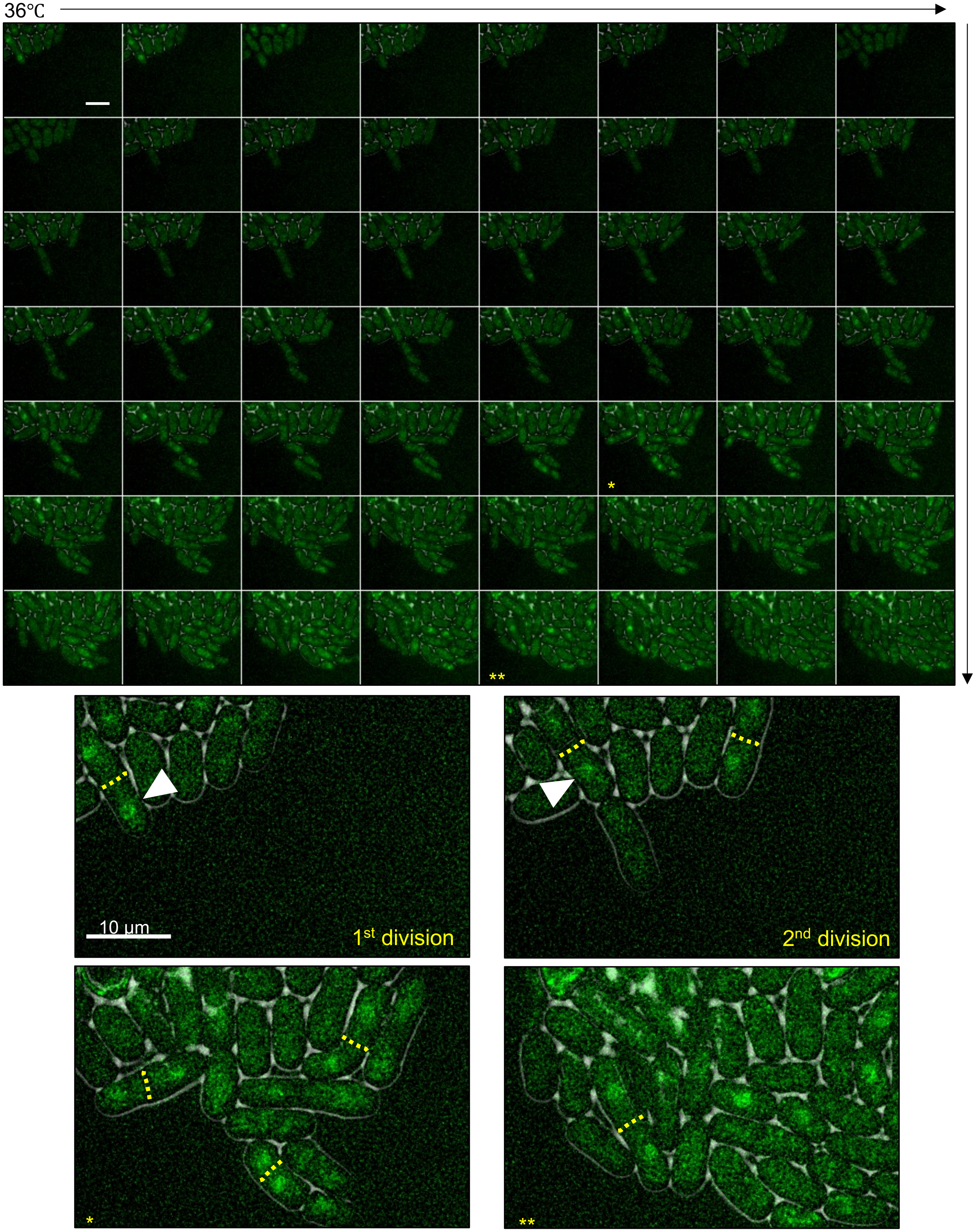

Supplementary Figure 5. WT cells show recurring nuclear Tos4 appearance in each cell cycle. Time-lapse images of WT cells at 36℃ for Tos4-GFP (FY10064). Arrowhead indicates Tos4. * and ** indicate presence of Tos4 after multiple divisions (enlarged on the bottom).

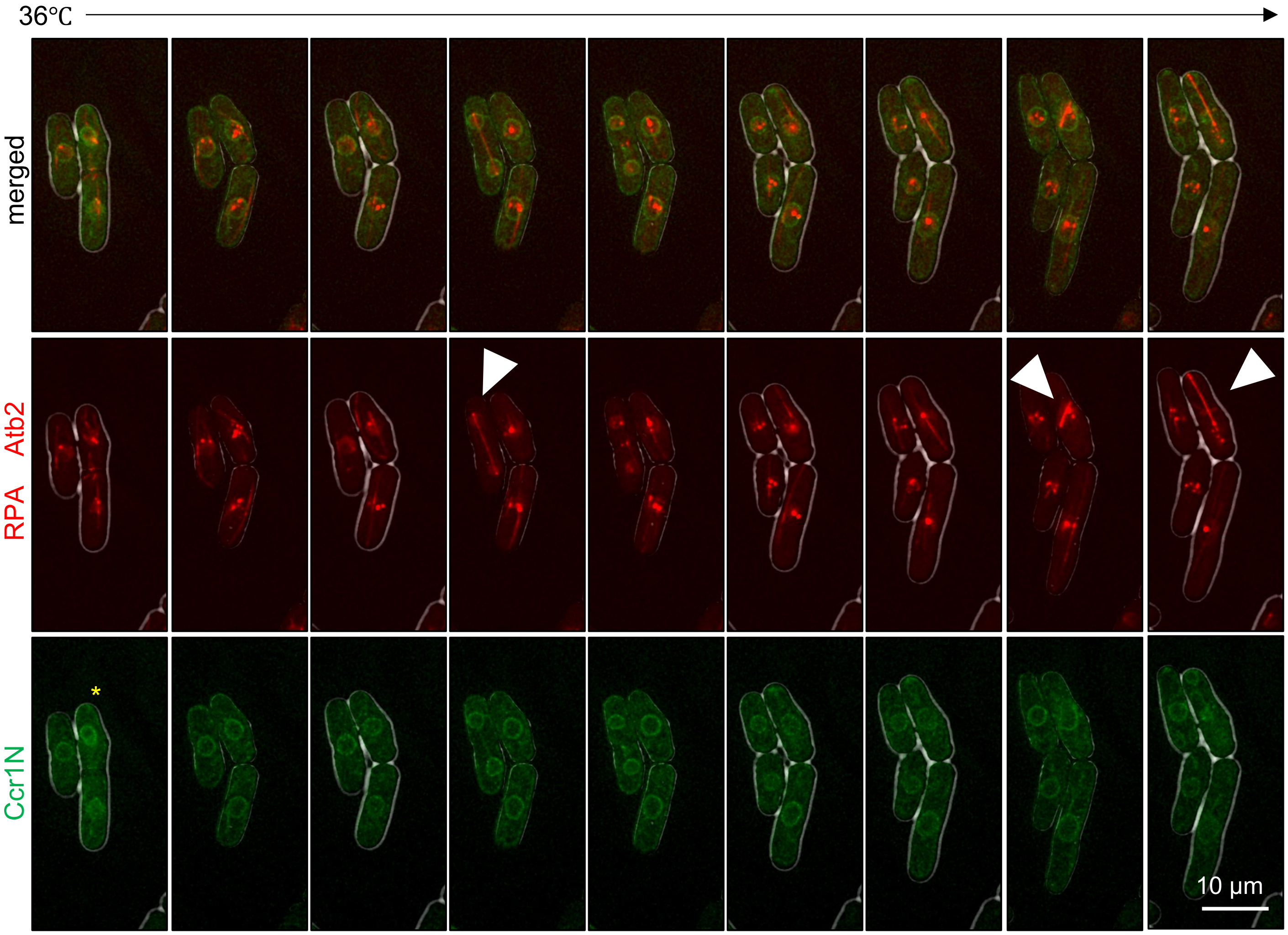

Supplementary Figure 6. *mcm4-dg* cells do undergo mitosis properly. Time-lapse images of *mcm4-dg* cells at 36℃ for RPA-mCherry, Atb2-mCherry, and Ccr1N-GFP (FY10092). Arrowhead indicates mitotic microtubules reorganization.

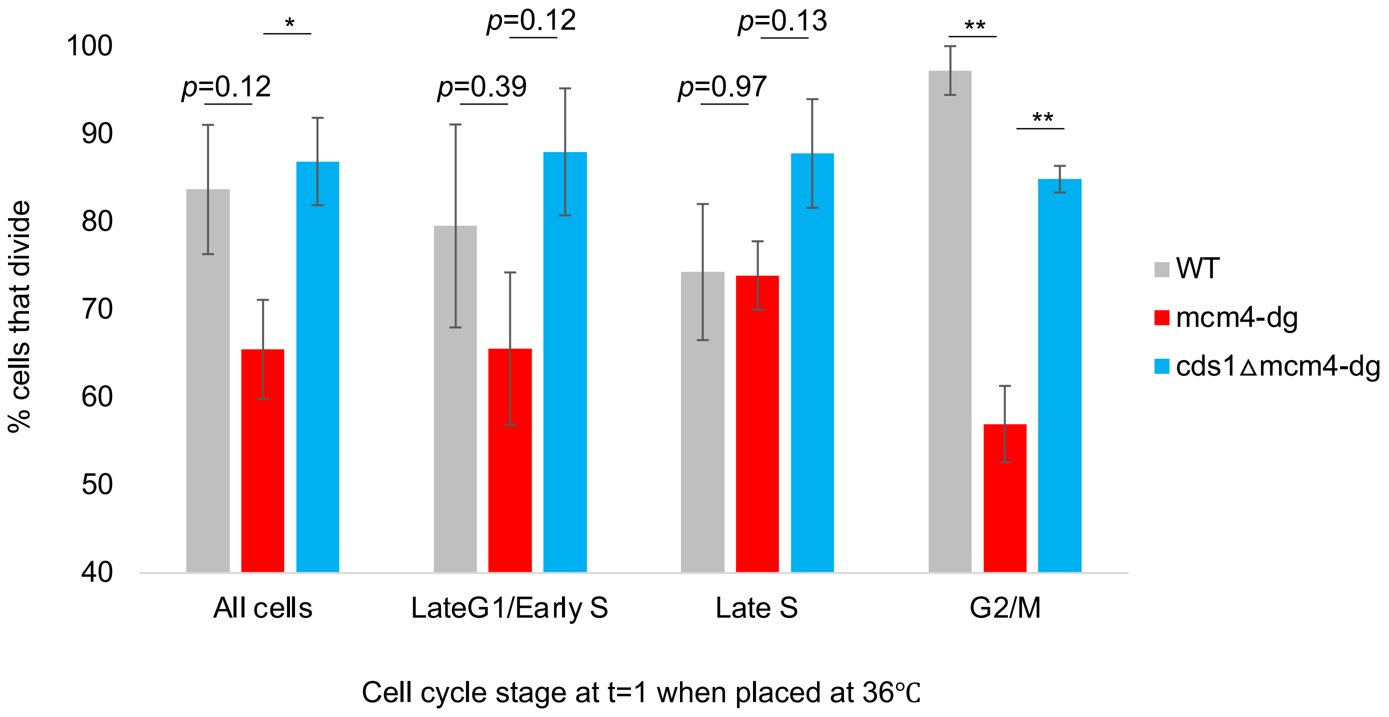

Supplementary Figure 7. Cds1 deficiency allows more *mcm4-dg* cells to enter cell cycle. Quantification of cells that divides at least twice when placed at restrictive temperature during timelapse imaging (6-8 h) (FY10064, FY10089, FY10098). Late G1/early S cells identified by presence of nuclear Tos4 in binucleate cells at t=1; Late S cells by nuclear Tos4 in mononucleate cells; G2/M cells by lack of Tos4 in mononucleate cells. * $P < 0.05$, ** $P < 0.01$

References in the Supplementary

Ahmed, S., B. Dul, X. Qiu, and N. C. Walworth, 2007 Msc1 acts through histone H2A.Z to promote chromosome stability in Schizosaccharomyces pombe. Genetics 177: 1487–1497.

Aronica, L., T. Kasparek, D. Ruchman, Y. Marquez, L. Cipak et al., 2015 The spliceosome-associated protein Nrl1 suppresses homologous recombination-dependent R-loop formation in fission yeast. Nucleic Acids Res. 44: 1703–1717.

Bjerling, P., R. A. Silverstein, G. Thon, A. Caudy, S. Grewal et al., 2002 Functional Divergence between Histone Deacetylases in Fission Yeast by Distinct Cellular Localization and In Vivo Specificity. Mol. Cell. Biol. 22: 2170–2181.

Gonzalez, Y., A. Saito, and S. Sazer, 2012 Fission yeast Lem2 and Man1 perform fundamental functions of the animal cell nuclear lamina. Nucleus 3: 60–76.

Maki, K., T. Inoue, A. Onaka, H. Hashizume, N. Somete et al., 2011 Abundance of prereplicative complexes (Pre-RCs) facilitates recombinational repair under replication stress in fission yeast. J. Biol. Chem. 286: 41701–41710.

Nakamura, K. I., A. Okamoto, Y. Katou, C. Yadani, T. Shitanda et al., 2008 Rad51 suppresses gross chromosomal rearrangement at centromere in Schizosaccharomyces pombe. EMBO J. 27: 3036–3046.

Noguchi, E., C. Noguchi, W. H. McDonald, J. R. Yates, and P. Russell, 2004 Swi1 and Swi3 Are Components of a Replication Fork Protection Complex in Fission Yeast. Mol. Cell. Biol. 24: 8342–8355.

Sanders, S. L., M. Portoso, J. Mata, J. Bähler, R. C. Allshire et al., 2004 Methylation of Histone H4 Lysine 20 Controls Recruitment of Crb2 to Sites of DNA Damage Histone H4 Methylation and Checkpoint Control Results Fission Yeast H4-K20 Methylation Is Set9 Dependent. Online 119: 603–614.

Ueno, M., T. Murase, T. Kibe, N. Ohashi, K. Tomita et al., 2004 Fission yeast Arp6 is required for telomere silencing, but functions independently of Swi6. Nucleic Acids Res. 32: 736–741.

Wolkow, T. D., and T. Enoch, 2003 Fission yeast Rad26 responds to DNA damage independently of Rad3. BMC Genet. 4: 1–11.
